# The future of the Black Sea: More pollution in over half of the rivers

**DOI:** 10.1007/s13280-022-01780-6

**Published:** 2022-09-08

**Authors:** Maryna Strokal, Vita Strokal, Carolien Kroeze

**Affiliations:** 1 Water Systems and Global Change, Wageningen University & Research, Droevendaalsesteeg 3a, 6708 PB Wageningen, The Netherlands; 2 National University of Life and Environmental Sciences of Ukraine, Heroiv Oborony 15, Kiev, 03041 Ukraine

**Keywords:** Future, Pollutants, Rivers, Scenarios, The Black Sea

## Abstract

The population in the Black Sea region is expected to decline in the future. However, a better understanding of how river pollution is affected by declining trends in population and increasing trends in economic developments and urbanization is needed. This study aims to quantify future trends in point-source emissions of nutrients, microplastics, *Cryptosporidium*, and triclosan to 107 rivers draining into the Black Sea. We apply a multi-pollutant model for 2010, 2050, and 2100. In the future, over half of the rivers will be more polluted than in 2010. The population in 74 sub-basins may drop by over 25% in our economic scenario with poor wastewater treatment. Over two-thirds of the people will live in cities and the economy may grow 9-fold in the region. Advanced wastewater treatment could minimize trade-offs between economy and pollution: our Sustainability scenario projects a 68–98% decline in point-source pollution by 2100. Making this future reality will require coordinated international efforts.

**Supplementary Information:**

The online version of this article (10.1007/s13280-022-01780-6) contains supplementary material, which is available to authorized users.

## Introduction

Surface water quality depends on human activities in drainage basins. In many world regions, population growth and urbanization are drivers of water pollution in the future (Jiang and O’Neill 2017; van Puijenbroek et al. 2019). It is expected that a growing population will generate more waste, leading to higher emissions of multiple pollutants to rivers in the future (Strokal et al. 2021b). In addition, the role of sewage in total river pollution is expected to increase because of rapid urbanization (Kc and Lutz 2017; Acuto et al. 2018). Southeast Asia is an example of such trends (Wang et al. 2020). However, the Black Sea region may show an opposite trend, because the population in certain countries is expected to decline in the future. The implications of this for water quality in the Black Sea are unknown.

**Fig. 1:**
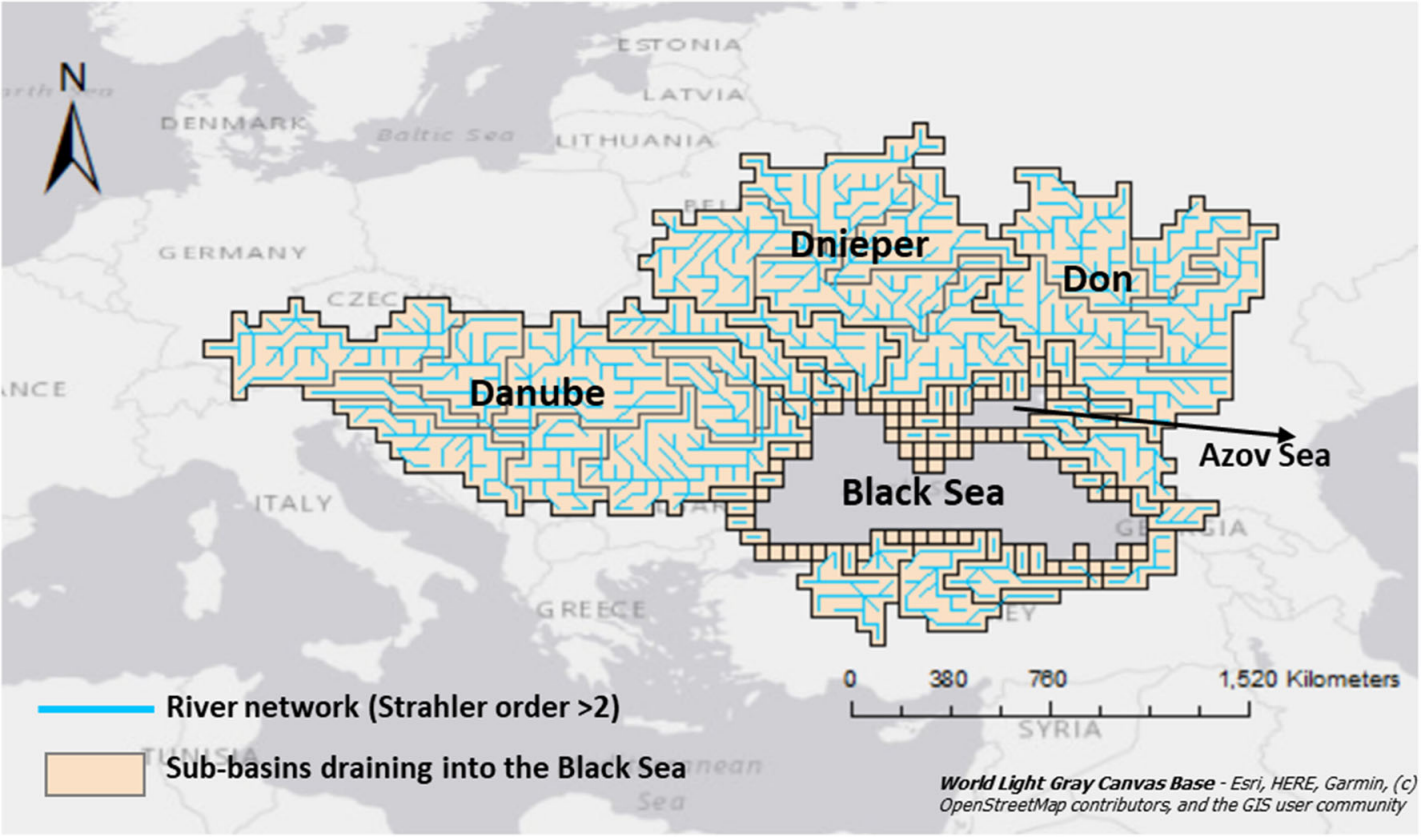
Rivers and their sub-basins draining into the Black Sea. *Source:* Strokal et al. (2019)

The Black Sea region is unique because of its location between Europe and South Asia (Text S1). Many rivers draining into the Black Sea are transboundary (Strokal 2021a, b). The drainage area includes 23 countries and 107 river sub-basins (Strokal et al. 2019) where 190 million people live (Fig. 1). It is a semi-enclosed sea, accumulating pollutants over time and it is a hotspot of different pollution types such as eutrophication, plastic pollution, and chemical contamination (BSC 2019).

Rivers transport urban pollutants to the Black Sea (Levent et al. 2018). The municipal wastewater is often collected by sewage systems (BSC 2019), that discharge the waste into rivers. Sewage effluents are point sources of nutrients (Strokal and Kroeze 2013; BSC 2019), pathogens (Hofstra et al. 2013; Aytan et al. 2019), plastics (Siegfried et al. 2017; Berov and Klayn 2020) and chemicals (Levent et al. 2018; Aytan et al. 2019) in rivers. Recent studies report on river pollution in the Black Sea region for specific areas such as southwest (Berov and Klayn 2020), southeast (Aytan et al. 2019), and northwest (Chugai et al. 2020). These studies typically focus on specific pollutants (Berov and Klayn 2020) and/or specific pollution sources (Chugai et al. 2020). A comprehensive analysis of water pollution from a multi-pollutant perspective is not available for the Black Sea. Moreover, future trends in river pollution are not well studied for multiple pollutants simultaneously, and for scenarios that account for expected trends in population, economy, and urbanization.

This study aims to quantify future trends in point-source emissions of nutrients, microplastics, *Cryptosporidium*, and triclosan to 107 rivers draining into the Black Sea. We account for future urbanization and socio-economic developments (e.g., population growth and economy). Our study focuses on the short-term (2010–2050) and long-term (2050–2100) future. Our focus on nutrients, microplastics, *Cryptosporidium* and triclosan is motivated by their impacts on nature and society and that these pollutants can be considered indicators of pressing and emerging water pollution problems (Strokal et al. 2021b; Li et al. 2022). Moreover, modeling approaches and data are available for these pollutants (see references in Fig. S1). We develop and evaluate a sub-basin version of the MARINA-Global model: Model to Assess River Inputs of pollutAnts to seAs (Strokal et al. 2021b) (Box S1; Tables S1–S5; Fig. S1,  “MARINA-Global model for the Black Sea” section). This model has been previously developed for nutrients, microplastics, *Cryptosporidium* and triclosan. We adjust the model for the rivers draining into the Black Sea. We apply three scenarios that reflect different trends in urbanization, socio-economic developments and the approaches toward wastewater treatment. We implement our scenarios into the model to analyze future trends.

## Materials and methods

### MARINA-Global model for the Black Sea

The existing MARINA-Global model (Strokal et al. 2021b) integrates modeling approaches for *Cryptosporidium* (Hofstra et al. 2013), nutrients (Mayorga et al. 2010; Strokal et al. 2016), microplastics (Siegfried et al. 2017), and triclosan (van Wijnen et al. 2017) for their point-source inputs to rivers. This is done for the world. Those modeling approaches were evaluated against measurements and using sensitivity analyses (see “Model evaluation and uncertainties” section for details).

We develop a new sub-basin version for 107 sub-basins of the Black Sea region for 2010, 2050 and 2100 (Fig. 1). These years are selected to represent the short-term (2010–2050) and long-term (2050–2100) pollution levels. The data and the model are available for these years (see Box S1 for equations). Our model is different from the original MARINA-Global model (Strokal et al. 2021b) in three aspects:

1. Our model calculates inputs of the pollutants using sub-basins as calculation units. The previous version of the MARINA-Global model used a 0.5° grid as a calculation unit (Strokal et al. 2021b). For the Black Sea, we include 107 sub-basins. We aggregated model inputs to those sub-basins draining into the Black Sea (Fig. S1; Tables S1, S2). We ran the model for 2010, 2050, and 2100 to quantify inputs of pollutants to the rivers in the sub-basins from point sources (Box S1). This means that our model is consistent in the spatial scale between the model inputs, calculation units and model outputs.
2. We split N and P into dissolved inorganic (DIN, DIP) and organic (DON, DOP) forms following the approach of Mayorga et al. (2010). The earlier version (Strokal et al. 2021b) focused on total N and P and did not split them into the forms (details are in Box S1; Fig. S1; Tables S1–S6). Focusing explicitly on dissolved inorganic and organic forms allows us to better understand their sources in rivers.
3. As a result, our model has been extended with more water quality variables. The earlier version (Strokal et al. 2021b) incorporated five water quality variables: N, P, *Cryptosporidium*, triclosan and microplastics. Our current model has seven: DIN, DON, DIP, DOP, *Cryptosporidium*, triclosan and microplastics. This implies that the multi-pollutant focus is expanded in our model for the Black Sea.

The model considers the following point sources of pollutants in rivers: sewage systems and open defecation. Sewage systems discharge N, P, *Cryptosporidium*, microplastics, and triclosan to rivers. N, P, and *Cryptosporidium* are from human waste that is collected by sewage systems in rural and urban areas. Microplastics in sewage systems result from the use of personal care products, laundry, household dust, and car tires. Triclosan in sewage is from the use of personal care products. The model quantifies inputs of these pollutants to rivers as a function of population (urban and rural), their connection rates to sewage systems, excretion or consumption rates of pollutants per capita, and the removal fractions of pollutants during wastewater treatment. Open defecation is a direct discharge of N, P, *Cryptosporidium* to rivers through human feces and urine (e.g., hanging toilets). The model quantifies inputs of these pollutants to rivers from open defecation as a function of the population (urban, rural) practicing open defecation and excretion rates of the pollutants per capita. Below, Eqs. 1–3 show the calculation method. Details with all equations are provided in Box S1.

1 $${\mathrm{RS}}_{\mathrm{i}.\mathrm{sb}}={\mathrm{RS}}_{\mathrm{dir}.\mathrm{i}.\mathrm{sb}}+{RS}_{\mathrm{sew}.\mathrm{i}.\mathrm{sb}},$$

2 $${\mathrm{RS}}_{{\mathrm{dir}}.{\mathrm{i}}.{\mathrm{sb}}}={\mathrm{Pop}}_{\mathrm{sb}}\cdot {\mathrm{fr}}_{{\mathrm{pop}}.{\mathrm{dir}}.{\mathrm{sb}}}\cdot {\mathrm{WShw}}_{{\mathrm{cap}}.{\mathrm{i}}.{\mathrm{sb}}},$$

3 $${\mathrm{RS}}_{{\mathrm{sew}}.{\mathrm{i}}.{\mathrm{sb}}}={\mathrm{Pop}}_{\mathrm{sb}}\cdot {\mathrm{fr}}_{{\mathrm{pop}}.{\mathrm{sew}}.{\mathrm{sb}}}\cdot {\mathrm{WShw}}_{{\mathrm{cap}}.{\mathrm{i}}.{\mathrm{sb}}}\cdot \left(1-{\mathrm{hw}}_{{\mathrm{frem}}.{\mathrm{i}}.{\mathrm{sb}}}\right),$$

where ${\mathrm{RS}}_{\mathrm{i}.\mathrm{sb}}$ is the annual input of pollutant *i* to rivers of sub-basins sb from all sources (sewage systems and open defecation). The unit is oocyst/year for *Cryptosporidium*, kg/year for nutrients, and microplastics, and g/year for triclosan. ${\mathrm{RS}}_{\mathrm{dir}.\mathrm{i}.\mathrm{sb}}$ is the annual input of pollutant *i* to rivers of sub-basin sb from open defecation resulting from all population. The unit is oocyst/year for *Cryptosporidium*, kg/year for nutrients, and microplastics, and g/year for triclosan. ${\mathrm{RS}}_{\mathrm{sew}.\mathrm{i}.\mathrm{sb}}$ is the annual input of pollutant *i* to rivers of sub-basin sb from sewage systems resulting from all population. The unit is oocyst/year for *Cryptosporidium*, kg/year for nutrients, and microplastics, and g/year for triclosan. ${\mathrm{Pop}}_{\mathrm{sb}}$ is the total population in sub-basin sb (people/year). ${\mathrm{fr}}_{\mathrm{pop}.\mathrm{dir}.\mathrm{sb}}$ is the fraction of the total population practicing open defecation as point source in sub-basin sb (0–1). ${\mathrm{fr}}_{\mathrm{pop}.\mathrm{sew}.\mathrm{sb}}$ is the fraction of the total population that is connected to sewage systems in sub-basin sb (0–1). ${\mathrm{WShw}}_{\mathrm{cap}.\mathrm{i}.\mathrm{sb}}$ is the excretion or consumption rate of pollutant *i* in sub-basin sb. The unit is oocyst/cap/year for *Cryptosporidium*, kg/cap/year for nutrients and microplastics, and g/cap/year for triclosan. The excretion rates are for N, P and *Cryptosporidium* in human waste (feces and urine). Consumption rates are for triclosan from personal care products and for microplastics from personal care products, car tyres, household dust and laundry. ${\mathrm{hw}}_{\mathrm{frem}.\mathrm{i}.\mathrm{sb}}$ is the fraction of removed pollutant *i* during treatment in sub-basin sb (0–1).

In our model, every pollutant has two pathways to enter rivers via sewage systems (see Eq. 2) and open defecation (see Eq. 2). The pathway of sewage systems involves pollutant removals during treatment. This is different for open defecation where human waste can enter rivers directly without treatment.

All data and its sources are in Fig. S1, and Tables S2–S5. Raw inputs for the model are grid-, country- and pollutant-specific and available from existing datasets (all references are in Fig. S1). Total, urban and rural population is available at the grid of 0.5° (Jones and O’Neill 2016). Fractions of urban and rural people connected to sewage systems and practicing open defecation are country-specific and were available from the Joint Monitoring Program (https://washdata.org/). This data was processed and provided from existing studies (Hofstra and Vermeulen 2016; Strokal et al. 2019). The removal efficiencies of pollutants during treatment were also available by country in existing studies (Van Drecht et al. 2009; Hofstra and Vermeulen 2016; Strokal et al. 2019). Pollutant-specific model inputs are excretion and consumption rates that were taken from existing studies for nutrients, *Cryptosporidium*, microplastics and triclosan (Mayorga et al. 2010; Hofstra and Vermeulen 2016; Siegfried et al. 2017; van Wijnen et al. 2017; Strokal et al. 2019). Data are provided in Tables S3–S5 and details about the sources of the data are in Fig. S1). We processed raw inputs to sub-basins using population. Table S2 explains how we did this. Model evaluation is described in “Model evaluation and uncertainties” section.

### Scenarios

**Fig. 2:**
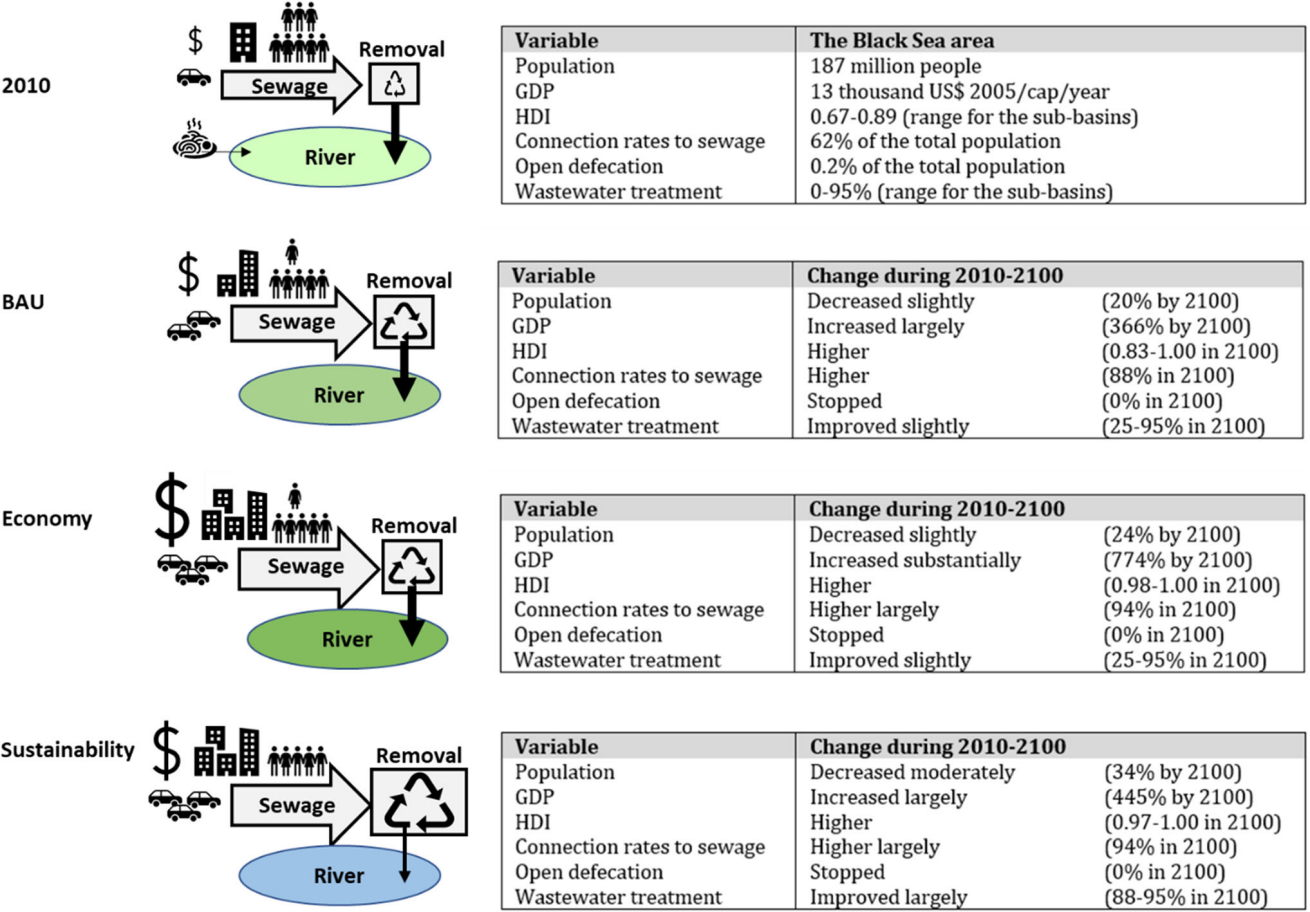
Summary of important indicators for the drainage area of the Black Sea for the year 2010 and three future scenarios (up to 2100): a Business as Usual (BAU), Economy and Sustainability scenarios. *GDP* is the gross domestic product at purchasing power parity, *HDI* is the human development index, *Population* is the total population in the drainage area of the Black Sea. GDP for 2010 is the average value over the Black Sea sub-basins. Values for the Human Development Index (HDI) and wastewater treatment efficiencies reflect the ranges for the sub-basins. Connection rates to sewage refer to the percentage of the total people that are connected to sewage systems in the drainage area of the Black Sea. The same holds for open defecation. References to the scenarios and their full descriptions are in Table S6 and Figs. S1–S4. Wastewater treatment efficiencies are in Figs. S5 and S6

We implemented three scenarios to analyze future pollution of rivers draining into the Black Sea based on the data from Strokal et al. (2021b) and Strokal et al. (2021a) (Fig. 2; Table S6). These are a Business as Usual (BAU), Economy, and Sustainability scenario. The three scenarios are selected to reflect different future trends in socio-economic development, urbanization, and wastewater treatment. The Economy and Sustainability scenarios assume high economic development and rapid urbanization, but differ in their approaches towards sustainability. The difference between the two can be used to analyze the effects of environmental policies under economic growth. The BAU scenario is selected to show the situation in the future following the historical trends, with a lower economic growth than the Economy scenario. BAU follows trends from Shared Socio-economic Pathway 2 (SSP, “middle of the road)”. The Economy and Sustainability scenarios are based on SSP5 and SSP1, respectively.

In our scenarios, the socio-economic development includes population (urban and rural), gross domestic products (GDP), and human development index (HDI). Urbanization includes connection rates of the population to sewage systems and wastewater treatment. Open defecation reflects sanitation. Population and GDP for 2050 and 2100 are based on SSP1 (for the Sustainability scenario), SSP2 (for the BAU scenario) and SSP5 (for the Economy scenario). Values for these model inputs are directly taken from existing studies (Fig. S1). Strokal et al. (2021b) interpreted quantitatively SSP storylines for connection rates of people to sewage systems, fractions of people practicing open defecation, wastewater treatment, and HDI for the countries. We aggregated these inputs to 107 sub-basins of the Black Sea (Tables S2, S6).

In the BAU scenario, the population and GDP trends follow SSP2 (Table S6). In 2010, 187 million people lived in the drainage area. By 2100, the population is projected to have decreased by 20%. HDI is expected to increase by 10% between 2010 and 2050 and further increase by 10% by 2100 (Fig. S2). GDP is expected to increase by 366% between 2010 and 2100 (Fig. S4). People will be richer and cities will have more sewage systems. As a result, the connection rates of people will increase, reaching 88% in 2100 (Fig. 2). Open defecation will stop and the use of P detergents will be forbidden (Fig. S4). Wastewater treatment will slightly improve in the future compared to 2010 (Fig. 2; Figs. S5, S6).

In the Economy scenario, trends in population and GDP follow SSP5 (Table S6). Between 2010 and 2100, the population is projected to decrease by 24% (Fig. 2). HDI is assumed to increase by 20% between 2010 and 2100 and further increase by another 20% by 2100 (Fig. S2). In contrast, GDP is projected to substantially increase by 774% by 2100 (Fig. 1; Fig. S3). Most people are expected to be connected to sewage systems (Fig. 2). Use of P detergents will be forbidden as in BAU (Fig. S4). Wastewater treatment will improve, but not largely compared to BAU and 2010 (Figs. S5, S6).

In the Sustainability scenario, the population and GDP trends follow SSP1 (Table S6). The population is also projected to decrease in the future (Fig. 2). GDP will increase by 445% between 2010 and 2100 (Fig. S3), leading to higher HDI in the future (Fig. S2). Most people will be connected to sewage systems. Open defecation and the use of P detergents will stop (Fig. S4). However, the wastewater treatment will improve largely, which is not in the other two scenarios (Figs. S5, S6). This is because in this scenario, supporting the Sustainable Development Goals (SDGs) is an important aspect. People will invest in implementing the best available today technologies to remove N, P, *Cryptosporidium*, triclosan, and microplastics in sewage influents. Examples of such technologies are efficient ultrafiltration for *Cryptosporidium* (Nasser 2016) and microplastics (Sun et al. 2019), and reverse osmosis for nutrients (Mondor et al. 2008) and microplastics (Sun et al. 2019).

## Results

### River pollution from point sources in 2010

**Fig. 3:**
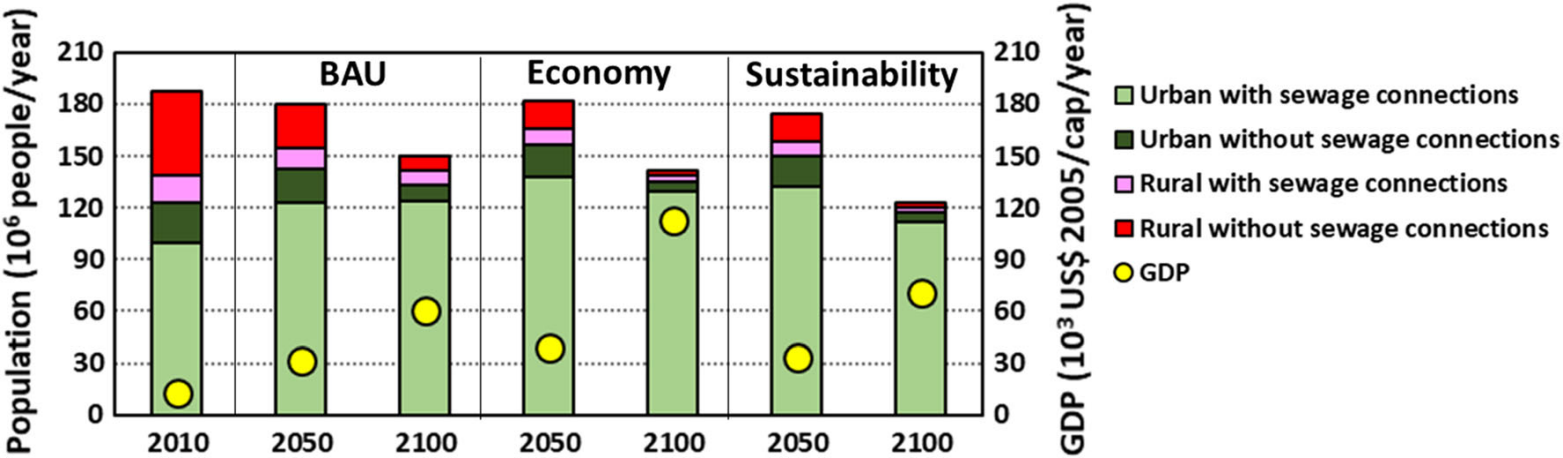
Future trends in the population (10^6^ people/year) and gross domestic products (GDP, 10^3^ US$ 2005/cap/year) in the drainage basin of the Black Sea. The drainage basin cover 107 sub-basins. Future trends are according to the Business as Usual (BAU), Economy and Sustainability scenarios. *Source:* see the “Materials and methods” section for the model and scenario descriptions (Box S1; Fig. 1; Figs. S1, S2; Tables S1–S6)

Rivers received 6.7 × 10^15^ oocysts of *Cryptosporidium*, 290 kton of total dissolved N (TDN), 73 kton of total dissolved P (TDP), 20 kton of microplastics, and 30 ton of triclosan (Fig. 3; Fig. S7). Almost all pollutants were from sewage systems and the remainder was from open defecation. Many northern sub-basins of the Danube and Dnieper received above 50 kg of TDN, 5 kg of TDP, 5 kg of microplastics, and 5 g of triclosan per km^2^ (Fig. 3). The same holds for most sub-basins in the south. In the east, the river pollution was generally lower than in the north and south (except for a few sub-basins, Fig. 3). Inputs of *Cryptosporidium* to rivers were generally higher for some downstream sub-basins of the Danube and Dnieper, and sub-basins in the south compared to the other sub-basins (Fig. 3). These results are the net effect of urbanization, economy, and treatment efficiencies (Figs. S5, S6, “MARINA-Global model for the Black Sea” and “Socio-economic drivers of water pollution” sections). For European sub-basins such as the Danube, important reasons were faster economic developments (HDI > 0.785, highest GDP) and higher population densities despite better wastewater treatment. The southern sub-basins in Asia were less economically developed (HDI < 0.785, lower GDP) than the Europeans, but had poorer treatment for most pollutants leading to more river pollution (Fig. 3; Fig. S5).

### Future pollution in the BAU scenario

**Fig. 4:**
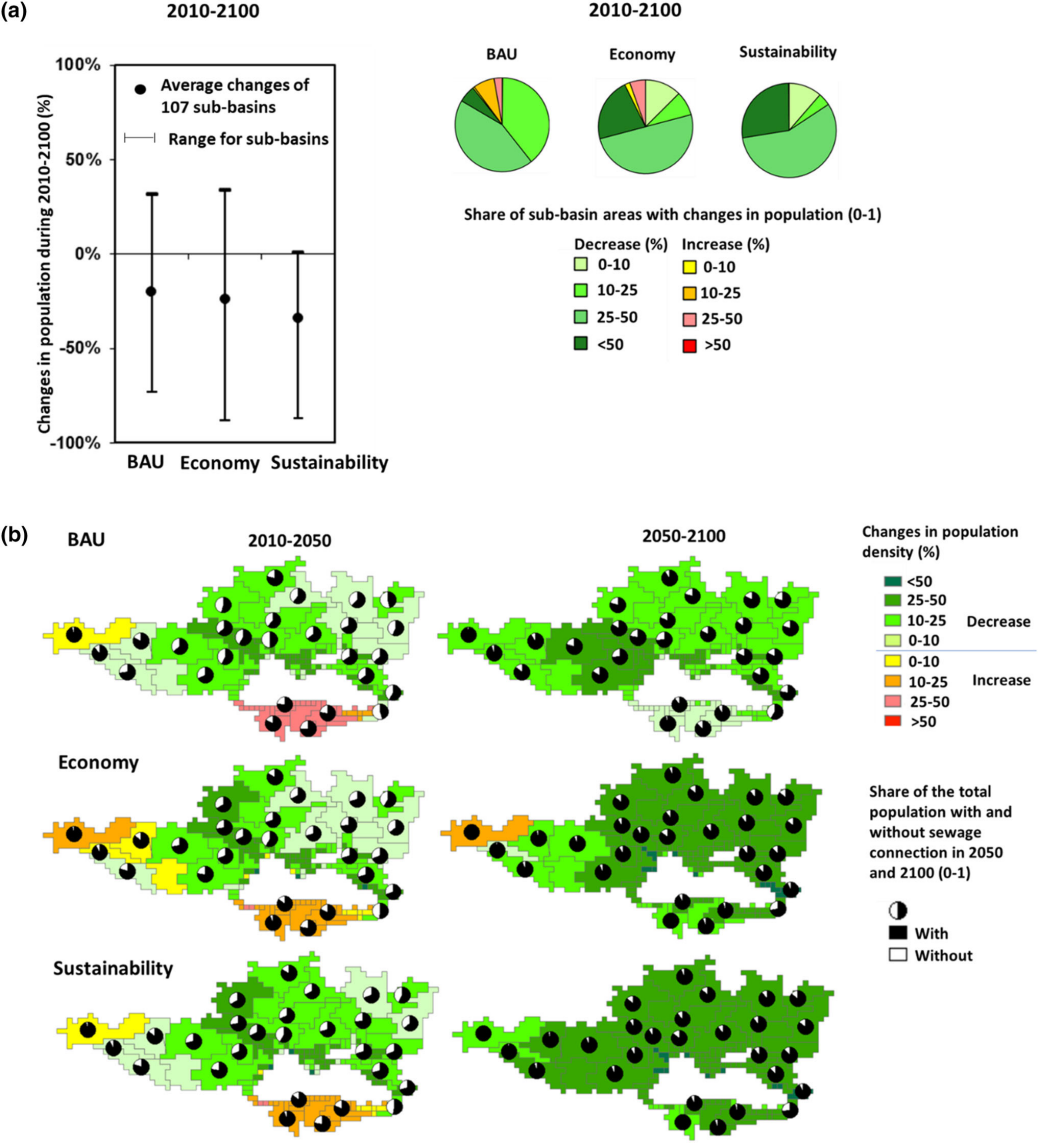
Future trends in the population for 107 sub-basins draining into the Black Sea. **a** Changes in the population during 2010–2100 (%) and the share of the sub-basin areas experiencing these changes. The average change of 107 sub-basins is calculated using the population from all sub-basins. **b** Changes in the population during 2010–2050 and 2050–2100 for 107 sub-basins. Future trends are according to the Business as Usual (BAU), Economy and Sustainability scenarios. The pies show the shares of the total population with and without sewage connections in 2010, 2050, and 2100. *Source:* see the “Materials and methods” section for the model and scenario descriptions (Box S1; Fig. 1; Fig. S2; Tables S1–S6)

River pollution in the drainage basin is projected to increase by 25% for TDN and 5% for microplastics during 2010–2100 (Fig. 4a). For the other pollutants, decreasing trends are calculated. However, river pollution differs among sub-basins. Almost 60% of the sub-basin areas will receive higher inputs of more than one pollutant in 2100 than in 2010 (Fig. 4b). These polluted sub-basins will accommodate 60% of the total population (Fig. 4c).

**Fig. 5:**
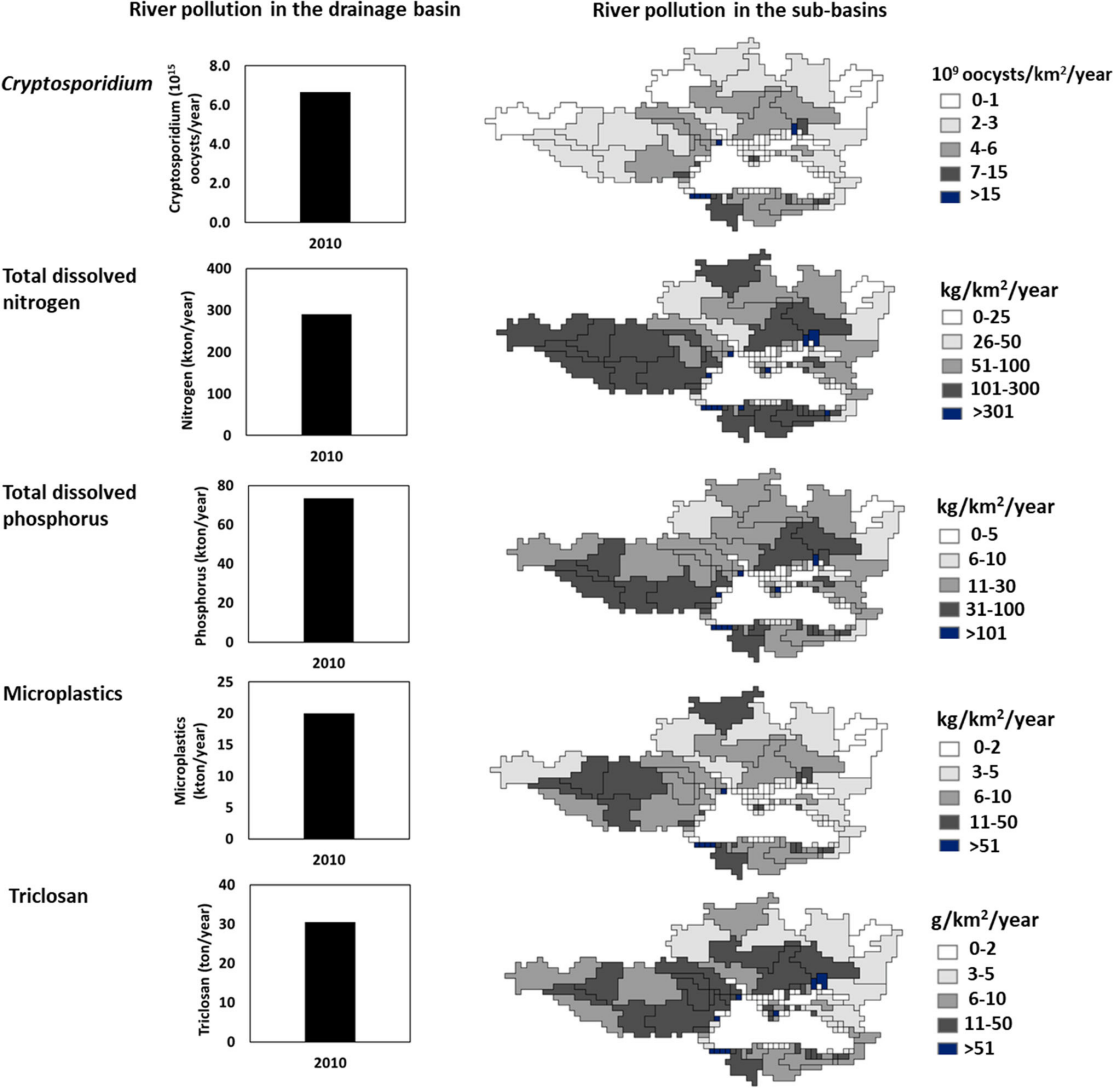
River pollution in the Black Sea basin in 2010. Graphs show annual point-source inputs of *Cryptosporidium*, total dissolved nitrogen (TDN), total dissolved phosphorus (TDP), microplastics, and triclosan to all rivers in the drainage area of the Black Sea (10^15^ oocysts/year for *Cryptosporidium*, kton/year for TDN, TDP and microplastics, ton/year for triclosan). Maps show annual point-source inputs of the pollutants to rivers at the sub-basin scale (10^9^ oocysts/km^2^ of the sub-basin area/year for *Cryptosporidium*, kg/km^2^ of the sub-basin area/year for TDN, TDP, and microplastics, g/km.^2^ of the sub-basin area/year for triclosan). Point sources include sewage systems and open defecation (direct discharges of untreated human waste to rivers). Figure S7 shows the total inputs of the pollutants to all rivers in 2050 and 2100. Changes in river pollution during 2010–2100, 2010–2050, and 2050–2100 are in Figs. 6 and 7. *Source:* the sub-basin scale MARINA-Global model (see the “Materials and methods” section for the model and scenario description)

**Fig. 6:**
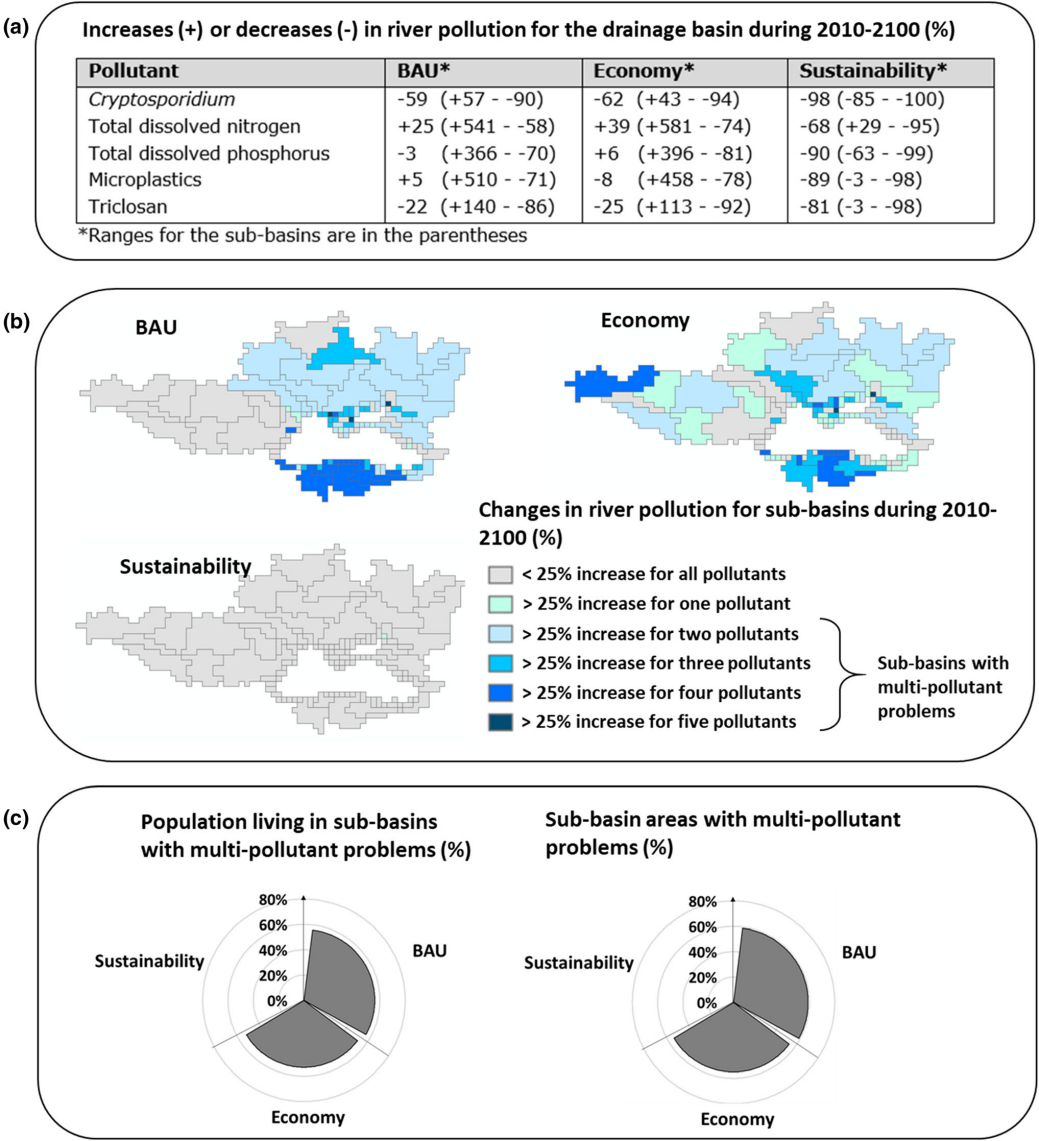
Changes in river pollution in the Black Sea basin during 2010–2100 (%). Future trends are based on the Business as Usual (BAU), Economy and Sustainability scenarios. **a** For the outside of the parenthesis, the table shows increases or decreases in annual point-source inputs of pollutants to all rivers in the Black Sea basin (% change relative to 2010). For the inside of the parentheses, the table shows the range for the changes in river pollution among sub-basins (% change relative to 2010). **b** Maps show the changes in river pollution for the individual sub-basins. The sub-basins are classified based on the number of pollutants for which we calculate higher or lower increases than 25% during 2010–2100 following (Strokal et al. 2021a, b). This threshold is arbitrary and does not reflect the ecological effects of the pollutants. However, this threshold gives an indication on the pollution directions: increases or decreases in the future. Thus, this threshold should be interpreted as the indication for rivers to become cleaner or more polluted in the future compared to the level of 2010. **c** A left pie shows the percentage of the population living in sub-basins with multi-pollutant problems in 2100. A right pie shows the percentage of the sub-basin area with multi-pollutant problems in 2100. River pollution is from point sources including sewage systems and open defecation (direct discharges of untreated human waste to rivers). *Source:* the sub-basin scale MARINA-Global model (see the “Materials and methods” section for the model and scenario description)

In the future, the northern rivers will be cleaner, but the southern and eastern rivers will be more polluted than in 2010 (Figs. 4, 5). Danube and Dnieper are the largest northern rivers (Fig. 1). Lower pollution levels are projected for *Cryptosporidium* in most sub-basins of the Danube and Dnieper during 2010–2050 and 2050–2100 (Fig. 5). This is because of a relatively high HDI (HDI > 0.785), leading to fewer infections (Fig. S2. Pollution with TDP and microplastics may increase by 2050 but then decrease by 2100 in many Danube and Dnieper sub-basins. These trends differ for TDN and triclosan (Fig. 5). Pollution trends are the net effect of the lower population (less waste production), improved wastewater treatment (especially for 2100), and high economic growth (Fig. 6; Figs. S3, S5, “Socio-economic drivers of water pollution” section).

The southern rivers may become more polluted in the future (Figs. 1, 5). During 2010–2050, > 50% increases are projected for four pollutants in the rivers (Fig. 5). This is a result of increasing trends in the population (Fig. 6; Fig. S5). By 2100, almost all southern rivers will receive more inputs of TDN, TDP, and triclosan than in 2050 despite decreasing trends in the population (Figs. 5, 6). Important reasons are growing economies (more nutrient excretion per capita) and hardly improved wastewater treatment (Figs. S3, S5, “Socio-economic drivers of water pollution” section).

For many eastern rivers (located in Georgia and Russia), increasing trends (> 25%) are projected for TDN, TDP, microplastics, and triclosan during 2010–2050 (Fig. 5). By 2100, river pollution with TDN and triclosan is projected to be higher than in 2050 (Fig. 5). This is despite a decline in the population during 2010–2050 and 2050–2100 (Fig. 6). Important reasons for higher pollution levels are growing economies and hardly improved wastewater treatment, which is similar to the southern rivers (Figs. S3, S5). By 2100, wastewater treatment may improve especially in the Russian sub-basins (Figs. S5, S6). However, it will not be enough to reduce inputs of TDN and triclosan to some rivers in 2100 to below the level of 2050 (Fig. 5).

### Future pollution in the Economy scenario

Population densities are projected to decrease in most sub-basins in the future like in the other two scenarios (see “MARINA-Global model for the Black Sea” and “Socio-economic drivers of water pollution” sections). However, the economy gets priority in this future (Figs. S2, S3). As a result, over half of the drainage area will experience multi-pollutant problems by 2100 (Fig. 4a–c). For this area, we calculate at least a 25% increase in river pollution with more than one pollutant during 2010–2100. During this period, river pollution in the drainage basin is projected to increase by 39% for TDN and 6% for TDP (Fig. 4a). For *Cryptosporidium*, triclosan, and microplastics, river pollution will decrease. However, these trends differ among sub-basins (Fig. 4a). River pollution with *Cryptosporidium* is projected to change from + 43% (increase) to − 94% (decrease) among sub-basins during 2010–2100. The range for TDN is from + 581% (increase) to − 74% (decrease). Large ranges among sub-basins are also calculated for TDP, microplastics, and triclosan (Fig. 4a).

**Fig. 7:**
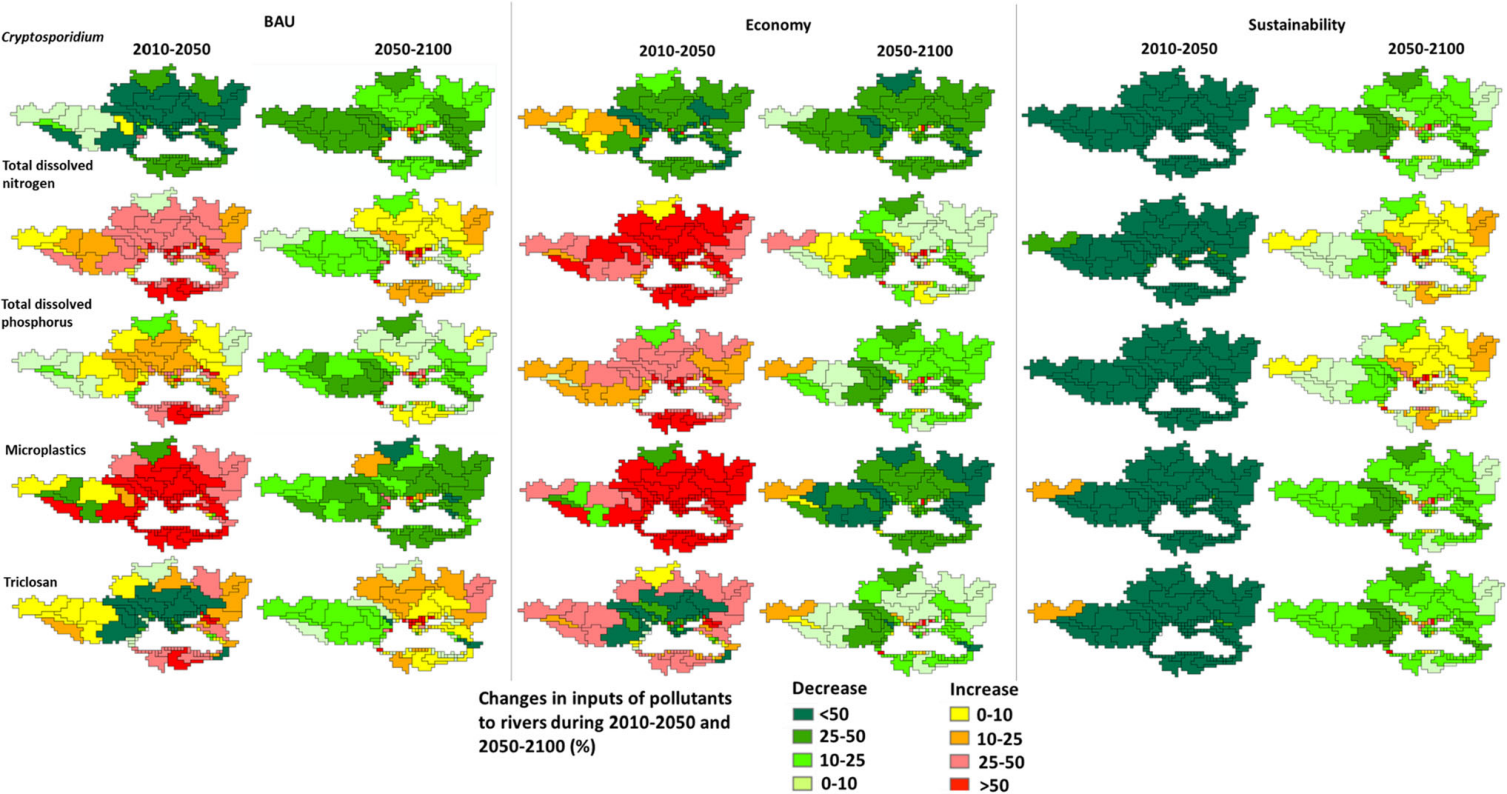
Changes in river pollution in the Black Sea basin during 2010–2050 and 2050–2100 (%). Maps show changes in annual point-source inputs of pollutants to rivers of the Black Sea at the sub-basin scale during the periods of 2010–2050 and 2050–2100 for the Business as Usual (BAU), Economy and Sustainability scenarios (%). *Source:* the sub-basin scale MARINA-Global model (see the “Materials and methods” section for the model and scenario description)

River pollution levels differ between 2050 and 2100 (Fig. 5). In 2050, river pollution in many northern, southern, and eastern sub-basins will receive > 50% more TDN and microplastics compared to 2010 (Fig. 7). In many southern sub-basins, inputs of TDP to their rivers are projected to increase by > 50% between 2010 and 2050. Increases by 25–50% during 2010–2050 are calculated for inputs of triclosan to many rivers of the Black Sea except for downstream sub-basins of the Danube and middle- and downstream sub-basins of the Dnieper. Inputs of *Cryptosporidium* to rivers are projected to increase by up to 25% between 2010 and 2050 for some sub-basins of the Danube during 2010–2050 (Fig. 5). By 2100, many rivers will receive fewer pollutants than in 2050. Exceptions are some sub-basins of the Danube and a few small rivers close to the coastal waters for which increases are projected by 2050. The reasons for the high river pollution in 2050 are largely associated with high economic growth (see “Socio-economic drivers of water pollution” section).

### Low pollution in the Sustainability scenario

In the future, the population and GDP trends are somewhat comparable with the other two scenarios (Figs. S2, S3). However, in this scenario, the priority is given to sustainable practices to support SDGs for cities (SDG11), clean water (SDG6), and a healthy economy (SDG8). As a result, advanced technologies will be implemented to treat wastewater. This scenario assumes the implementation of tertiary technologies (e.g., ultrafiltration methods, reverse osmosis, constructed wetlands) that can remove more than 75% of the five pollutants from the wastewater in sewage systems according to the existing studies (Jaffer et al. 2002; Kristensen et al. 2004; Foged et al. 2012; Pérez-González et al. 2012; Lutchmiah et al. 2014; Tervahauta et al. 2014; Xie et al. 2016). This is the most important difference between this scenario from the other two.

River pollution in the drainage basin is projected to decrease by 68% for TDN, 81% for triclosan, 89% for microplastics, and 98% for *Cryptosporidium* in 2100 compared to 2010 (Fig. 4). Cleaner rivers are projected for most sub-basins (Figs. 4, 5). A few sub-basins (e.g., upstream Danube) may still have 10–25% more inputs of microplastics and triclosan in their rivers in 2050 (Fig. 5). This is the net effect of faster economic development (for microplastics) and higher population density (for microplastics and triclosan). Rivers of some sub-basins are projected to have higher inputs of TDN and TDP in 2100 than in 2050 (Fig. 5). This is mainly because of higher protein-rich food. These are the southern sub-basins, upstream of the Danube, and some sub-basins of Dnieper and Don in the north and east (Fig. 5). Furthermore, a few small rivers along the northern coast may receive more inputs of all five pollutants in 2100 compared to 2050. This indicates the development of the coastal regions in the long future.

## Discussion

### Model evaluation and uncertainties

We follow the “building trust” approach (Strokal 2016) to evaluate both model inputs and outputs. This approach was developed for large-scale water quality models for which validation is challenging (Strokal et al. 2021b, 2022; Li et al. 2022). The approach is meant to evaluate the model. This evaluation consists of several options in which validation is only one of them. This implies that the “building trust” approach takes a broader perspective in model evaluation and goes beyond the typical model validation. This is because the “building trust” approach helps to better understand the uncertainties and build trust in models that are not easily validated. It includes options for model inputs (expert knowledge, sensitivity analysis), modeling approaches (model comparisons), and model outputs (validation, model comparisons). In our study we apply three options that are elaborated below.

#### Option 1 is to evaluate the modeling approach and model outputs

Option 1 is to evaluate the modeling approach and associated model outputs. Our model integrates the following, evaluated, existing modeling approaches for individual pollutants: Global NEWS-2 for nutrients (Mayorga et al. 2010), GloWPa for *Cryptosporidium* (Hofstra et al. 2013), the TCS model for triclosan (van Wijnen et al. 2017), and the microplastic model (Siegfried et al. 2016). The Global NEWS-2 model was validated for the Black Sea in the previous study (Strokal and Kroeze 2013). That study calculated Pearson’s coefficient of determination (*R*^2^) using observations of nutrients at the river mouth. Observations were available for around 2000 for eight rivers: the Danube, Dnepr, Don, Dniester, Bug, Kuban, Sakarya and Yesil (see the references for observations in Strokal and Kroeze 2013). Validation results showed a good performance of the model (*R*^2^ = 0.77) for nutrients. The other models (GloWPa, TCS and microplastics) were evaluated with a comprehensive sensitivity analysis (Hofstra et al. 2013) and/or against observations (Siegfried et al. 2017; van Wijnen et al. 2017; Vermeulen et al. 2019) for a set of rivers. For example, the microplastic model was evaluated for individual rivers including the Danube. The model of Siegfried et al. (2017) calculated around 1.5 kton of microplastics exported by the Danube per year, which was close to the estimate of Lechner et al. (2014). The sensitivity analysis of Hofstra et al. (2013) showed the importance of human waste and treatment in *Cryptosporidium* emissions.

Our modeling approaches for nutrients, triclosan, microplastics and *Cryptosporidium* are based on those previously evaluated models. However, our model misses other pollution sources (Strokal and Kovpak 2021) such as agriculture (Wen et al. 2017; Mogollón et al. 2021) and aquiculture (Wang et al. 2019). Diffuse sources such as runoff from agricultural fields can play an important role in the total pollution level of nutrients in rivers (Mogollón et al. 2021). Mismanaged waste on land can be an important diffuse source of macroplastics in rivers (Lebreton et al. 2017; Meijer et al. 2021). Microplastics can result from agricultural mulching in crop production (Huang et al. 2020). Macroplastics can be broken down into microplastics in rivers (van Wijnen et al. 2019). Thus, our model may underestimate the pollution levels for today, but not likely for urban areas where sewage systems dominate. This is the case for many sub-basins of the Black Sea region (Figs. 6, 7).

#### Option 2 is to compare with other studies

Option 2 is to compare our model outputs with existing modeling studies. We compare point-source inputs of pollutants to rivers with earlier studies (see Table 1; Table S7). Our results for N and P are in the range of the existing modeling studies (Table 1; Table S7). For example, annual inputs of total N from point sources to all rivers draining into the Black Sea are 0.42 Tg in our study. The other existing studies estimate between 0.35 and 1.3 Tg/year (see Table 1; Table S7). For total P, our estimate is 0.07 Tg/year, which is close to the existing studies of 0.07–0.15 Tg/year (see Table 1). Our inputs of DIN, DON, DIP, and DOP to individual rivers from point sources are somewhat higher (e.g., Danube for DIN, and DON) or lower (e.g., Don) compared to Global NEWS-2 (Table 1; Table S7). For the Danube, our estimate is 95 kton of DIN and 23 kton of DON in the river. These values are 77 kton for DIN and 19 kton for DON according to Global NEWS-2. In contrast, the Don River receive 23 kton of DIN and 8 kton of DIP in 2010 according to our model, which is lower than in Global NEWS-2 (32 kton for DIN and 11 kton for DIP, Table 1; Table S7). The differences between our and Global NEWS-2 models are because the models differ in time and space. We focus on the year 2010. Global NEWS provided estimates for the year 2000. Between 2000 and 2010, the socio-economic developments may have changed, leading to higher or lower pollution levels in 2010. Another reason is that we consider more rivers than Global NEWS-2. The drainage areas of our rivers were delineated using land masks and water flows from the VIC hydrological model (Markert 2017), which was different in Global NEWS-2 (the WBM hydrological model Fekete et al. 2010). In addition, the model considers open defecation, which is ignored in Global NEWS-2 (Mayorga et al. 2010). For microplastics and triclosan, our model results are generally in line with previous studies (Table 1; Table S7). We calculate 20 kton of microplastics in all rivers, which is close to the estimate of Siegfried et al. (2017). For the Danube River, our value is 8720 kton for the year 2010. This is close to the values of 8854 kton/year and 9031 kton/year from the other studies (see Table 1; Table S7 for references).

**Table 1:** Inputs of pollutants to rivers of the Black Sea region in this study and according to the existing studies. Letters (a)–(g) refer to the references of the other studies. These references can be found in Table S7

	This study	Other studies (Table S7)
Dissolved inorganic nitrogen in rivers (kton/year)		
Dnieper	33	30^a^
Danube	95	77^a^, 30–55^b^
Dniester	5	4^a^
Don	23	32^a^
Bug	2	4^a^
Kuban	3	8^a^
Sakarya	10	10^<a^
Yesil	4	0^a^
Dissolved inorganic phosphorus in rivers (kton/year)		
Dnieper	11	10^a^
Danube	29	24^a^, 20–30^b^
Dniester	2	1^a^
Don	8	11^a^
Bug	0.7	0.4^a^
Kuban	1	3^a^
Sakarya	3	4^a^
Yesil	1	0^a^
Dissolved organic nitrogen in rivers (kton/year)		
Dnieper	9	8^a^
Danube	23	19^a^
Dniester	2	1^a^
Don	6	8^a^
Bug	1	1^a^
Kuban	1	2^a^
Sakarya	3	3^a^
Yesil	1	0^a^
Dissolved organic phosphorus in rivers (kton/year)		
Dnieper	0.11	0.10^a^
Danube	0.31	0.24^a^
Dniester	0.05	0.01^a^
Don	0.09	0.11^a^
Bug	0.01	0.01^a^
Kuban	0.01	0.03^a^
Sakarya	0.03	0.04^a^
Yesil	0.01	0^a^
Total nitrogen and phosphorus in all rivers (Tg/year)		
Nitrogen	0.42*	0.35^a^, 0.48^c^, 0.6–1.3^d^
Phosphorus	0.07*	0.07^a^, 0.09^c^, 0.08–0.15^d^
Microplastic inputs to rivers (kton/year)		
All rivers	20	25^e^
Danube	8720	8854^e^, 9031^f^
Triclosan inputs to rivers (g/km^2^/year)		
Danube	13.8	11.7^g^

*Total nitrogen and phosphorus inputs to all rivers of the Black Sea region from sewage systems and open defecation (direct inputs of human waste to rivers). These inputs are total nutrients

Our model outputs reflect five pollution types. However, rivers might also experience other pollution types. For example, rivers might be polluted with organics (Wen et al. 2017) and other chemicals (Acuña et al. 2020). In this study, five pollution types serve as illustrative examples that show the impact of urban-related drivers. Future studies can build on this and add more pollutants to the model. We model inputs of pollutants to rivers. We do not model biogeochemical interactions between the pollutants in the rivers. This is outside the scope of our research. However, biogeochemical interactions might be important to consider in pollution control. Our study provides a multi-pollutant tool that opens an opportunity to add biogeochemical interactions in the future research.

#### Options 3 is to perform the sensitivity analysis

**Fig. 8:**
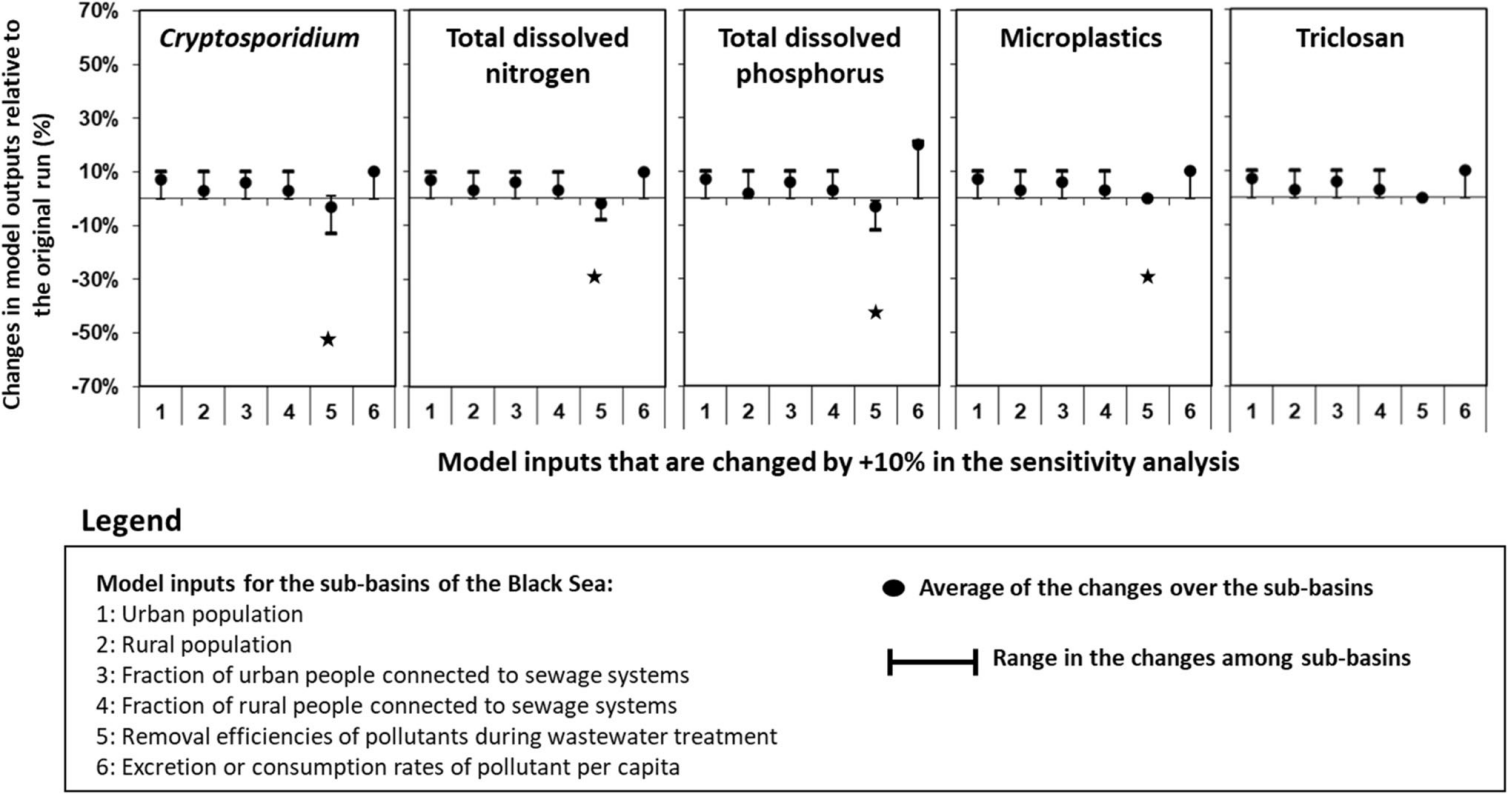
Results of the sensitivity analysis. Graphs show changes in the model outputs relative to the original run for 2010 (%). These changes are resulted from changed model inputs by + 10%. Model outputs are annual inputs of the five pollutants to rivers in the sub-basins of the Black Sea. Model outputs for the upstream sub-basins of Danube (for *Cryptosporidium*, nitrogen, and phosphorus) and Dnieper (for microplastics) are shown with the start in the graphs. For these sub-basins, changes in model outputs range from 33% (microplastics) to 60% (*Cryptosporidium*) (Tables S8, S9). These are the transboundary sub-basins. Larger sensitivities for these sub-basins illustrate the importance of wastewater treatment in pollution control

Option 3 is to better understand the sensitivity of model outputs to changes in uncertain model inputs (Fig. 8). We downscaled or upscaled inputs from existing datasets (Box S1; Fig. S1; Tables S1–S6). The removal efficiencies and excretion rates are uncertain. They can vary considerably across scales and pollutants. In our model, we estimate the removal efficiencies using the population that has primary, secondary, and tertiary treatments (sub-basin specific from existing studies, Fig. S1) and their removal efficiencies for each pollutant (Fig. S1). Our removal rates are averaged values for the sub-basins. The same holds for the excretion rates. Thus, we performed a sensitivity analysis. We changed the following model inputs by + 10%: urban and rural population, fractions of the urban and rural population connected to sewage systems, removal efficiencies, and excretion or consumption rates of pollutants (Fig. 4; Tables S3, S9). For this, we ran the model 30 times separately for five pollutants and six model inputs. Then, we compared the results of the model runs with the original model run. For this, we calculated the differences that are shown in Fig. 8.

Results show relatively small sensitivity of the model outputs to changes in those inputs (Fig. 8). In general, the model outputs are relatively more sensitive to changes in the removal efficiencies and excretion rates compared to the other model inputs. For example, exceptions are a few rivers where changes in model outputs exceed 10%, especially for the removal efficiencies. Changes in model outputs for the upstream sub-basins of Danube (for *Cryptosporidium*, nitrogen, and phosphorus) and Dnieper (for microplastics) range from 33% (microplastics) to 60% (*Cryptosporidium*) (Fig. 8). This might be associated with the transboundary aspects. In general, increasing removal efficiencies by 10% results in lower river pollution with the pollutants (Fig. 8). This is the opposite effect compared to the effect of the other model inputs. This illustrates the importance of wastewater treatment in pollution control. Thus, we believe that these insights can support our main messages for point sources of multiple pollutants in rivers. Our sensitivity analysis is based on the 10% change in the model inputs. The previous study (Strokal et al. 2021b) used the 50% change and found similar conclusions.

The three abovementioned options help us to better understand the sources of the uncertainties and their implications for the main results. Uncertainties are largely associated with our steady-state modeling approach (Options 1–2), processing model inputs (Option 3), and scenario assumptions. The steady-state modeling approach does not consider dynamics and legacy effects. These are important aspects to consider in studies on seasonality and river exports. Our study focuses on annual, long-term trends (2010–2100) in emissions of pollutants to rivers from sewage and open defecation. The other source of uncertainties is associated with our scenarios, which should not be interpreted as predictions. Rather, we aim to explore possible futures according to the BAU, Economy, and Sustainability trends. This scenario analysis is widely used in water quality assessments (van Puijenbroek et al. 2019). We realize that our assumptions can be debatable. For example, we assumed that advanced technologies for wastewater treatment will be implemented in all sub-basins of the Black Sea region in the Sustainability scenario. This is an optimistic assumption considering the current challenges (e.g., COVID-19 impacts, political instabilities, etc.). However, it is not an impossible scenario for the year 2100. We assume that implementations of advanced technologies can be possible considering the long-term future. Thus, the scenario results provide useful insights into future river pollution (“Results” section).

### Lessons learned from this study

The new insights learned from our study are associated with the modeling approach and the results. We present a new version of the multi-pollutant model for 107 sub-basins draining into the Black Sea (see “MARINA-Global model for the Black Sea” section). Our model is consistent in the sub-basin scale between the model inputs, calculation units and model outputs for 2010, 2050 and 2100. Our model has been extended with more water quality variables compared to the original version (MARINA-Global Strokal et al. 2021b). We focus on DIN, DIP, DON, DOP, *Cryptosporidium*, triclosan and microplastics. The choice for these pollutants is justified by their impacts on nature and society as well as the availability of data (see “Introduction” section and Fig. S1). We realize that many more pollutants exist in water (Ippolito et al. 2015; Acuña et al. 2020; Tramoy et al. 2020). However, our chosen variables allow us to simultaneously assess multiple impacts of water pollution. Examples are nutrient-induced eutrophication and C*ryptosporidium*-induced diarrhea. Our model takes a multi-pollutant perspective. Such a tool is useful in exploring ways to reduce multiple pollutants simultaneously to avoid pollution swapping. Our tool can be expanded with more pollutants, and it can be applied to other regions in the world.

Our results show the impact of socio-economic developments on inputs of DIN, DON, DIP, DOP, *Cryptosporidium*, triclosan and microplastics to rivers from urban areas. We show that the population is expected to decline, but income levels are projected to increase considerably in the future (“Socio-economic drivers of water pollution” section). In addition, urbanization (more cities and sewage systems) is expected to rapidly increase in the future. As a net effect, more river pollution is projected for the future. These trends differ from those in on many other world regions. An example is Africa where the population is expected to increase fast in the coming years (O’Neill et al. 2017). More people will lead to more waste in cities and thus more pollutants in rivers (Strokal et al. 2021b). Thus, the main driver for increasing water pollution in Africa is the fast-growing population. This is different for the Black Sea region where the economy is a more important factor in pollution control. Another example is China, where the population is expected to stabilize, but the economy will grow (Wang et al. 2020).

We focus on the short- (2010–2050) and long-term (2050–2100) futures. This helps to better understand the causes of water pollution in the near and far future. For example, in the short-term (2010–2050), water pollution is expected to be higher in many rivers and for most of the studied pollutants in the BAU and Economy scenarios (Fig. 5). This is different for the period of 2050–2100 when decreases in inputs of most pollutants are calculated for many rivers (Fig. 5). Earlier studies for the Black Sea often focus on either individual pollutants, rivers, or periods (Aytan et al. 2020; Berov and Klayn 2020; Chugai et al. 2020; Gonzalez-Fernandez 2020; Korshenko et al. 2020; Miladinova et al. 2020; Nacar et al. 2020). Our study is thus more complete.

### Socio-economic drivers of water pollution

Water pollution levels can be explained by the socio-economic drivers in the drainage basin of the Black Sea. There are considerable socio-economic differences among sub-basins draining into the Black Sea (Figs. 6, 7; Figs. S2, S3). When studying point-source pollution in rivers, there are a few important drivers: demography, urbanization, sewage connection, and wastewater treatment (Strokal et al. 2021b). These in turn depend on economic growth and human developments. Today, urbanization is moderate, and economic development is relatively low especially in the northern, eastern, and southern sub-basins when compared to the western European sub-basins. In 2010, around 190 million people lived in the drainage area of the Black Sea, of which 62% were connected to sewage systems (Fig. 6). About half of the total population was urban. GDP was 13 000 US$ 2005 per capita. However, this differs among sub-basins (Fig. S3). In 2010, the population density was generally higher in the western and northern parts of the Black Sea basin (e.g., the European sub-basins of the Danube) compared to the eastern and southern parts (e.g., the sub-basins of the Azov Sea and Turkey). More people were generally connected to sewage systems in the western (e.g., the Danube) than in the eastern part of the Black Sea basin (e.g., Asian sub-basins) in 2010 (Figs. 6, 7).

By 2050, the scenarios project fewer people, but much larger economies for many sub-basins. The population will decrease by 3–7%, but GDP will grow up to 3-fold in the drainage basin depending on the scenario (Fig. 7). Over two-thirds of the total population will be urban and connected to sewage systems in 2050. Trends differ among sub-basins: the population decreases from 1 to 60% and GDP increases from 1.4- to 9.6-fold in the scenarios during 2010–2050 (Fig. 6; Fig. S3). Exceptions are the Danube upstream and southern sub-basins for which we calculate increases in the population by up to 50% (Fig. 6).

By 2100, the population in 74 sub-basins may drop by over 25% in our economic scenario (Fig. 6a). These sub-basins occupy around two-thirds of the drainage basin. During 2010–2100, the population in the drainage basin is projected to increase by 20–34% depending on the scenario (Fig. 6a). GDP will increase by a factor of 4.7–5.5 in the BAU and Sustainability scenarios, and by a factor of 9 in the Economy scenario (Fig. 7; Fig. S3). Future trends differ among sub-basins (Fig. 6). For around half of the sub-basin areas, we calculate at least a 25% decrease in the population during 2010–2100 in BAU. In the Economy and Sustainability scenarios, this decrease is calculated for over two-thirds of the sub-basin area (Fig. 6a). For GDP, increases range from 2- to 24-fold among sub-basins during 2010–2100 (Fig. S3). During 2050–2100, the population densities are projected to decrease in many sub-basins. Most of the population will be urban and connected to sewage systems in 2100 (Fig. 6).

### Minimizing trade-offs between economic growth and water pollution

Our study shows that river pollution from urban activities may increase in the future, despite a declining population in the Black Sea region (“Results” and “Socio-economic drivers of water pollution” sections). Clearly, there are trade-offs between economic growth and urban water pollution. Economic growth implies that people may have more income, which is projected in our scenarios for the period of up to 2100. Our Economy scenario shows the trade-off between economic growth and water pollution. In this scenario, income levels are projected to be higher than in 2010 (see “Socio-economic drivers of water pollution” section). Higher-income levels may allow people to produce and access more food (leading to more nutrients in human waste), expand sewage systems (leading to more waste in treatment facilities), or buy more cars (leading to the production of microplastics) than in the past. To minimize these trade-offs, improving wastewater treatment is important. Our Sustainability scenario illustrates the importance of implementing advanced technologies to reduce urban water pollution in the period up to 2100 under the economic growth. This scenario indicates that the economy in this region can develop with low environmental impacts. For urban areas, advanced technologies play an important role. Examples of such technologies are ultrafiltration methods (Nasser 2016; Sun et al. 2019), reverse osmosis (Mondor et al. 2008), stabilization ponds, and constructed wetlands (Vymazal 2007). Some of the technologies can remove more than one pollutant, reducing multi-pollutant problems in urban rivers (Strokal et al. 2021b).

The BAU projections are not so optimistic about the effectiveness of wastewater treatment in the Black Sea region. In our Sustainability scenario, on the other hand, full implementation is assumed of advanced wastewater technologies that are already known today, but not yet widely implemented (Halden and Paull 2005; Thompson et al. 2005; Butler et al. 2012; Chen et al. 2012; Nasser 2016; van Puijenbroek et al. 2019). As a result, most point-source pollution of rivers can be 68–98% lower in 2100 than today. However, several of the advanced technologies are still in the experimental or pilot phase and need upscaling. Achieving this may seem optimistic in the near future, considering the required capacities of treatment facilities and urban infrastructure. Nevertheless, we consider that by 2100 full implementation of these already existing technologies is feasible (Strokal et al. 2021b, 2022). Our study of the Black Sea rivers can be relevant for other regions with urbanization and economic growth. Our sub-basin model can be used for future analyses of rivers in other world regions.

Our modeling approach shows how high economic growth may increase point-source inputs of multiple pollutants to rivers (as in our Economy scenario). Our multi-pollutant approach differs from many other studies focusing on single pollution types such as eutrophication (Borysova et al. 2005; Selman et al. 2008; Gao and Zhang 2010; van Wijnen et al. 2015; Lazăr et al. 2018; Strokal et al. 2020). We account for interactions between population, economic activities, and multiple pollutants. Economic growth often implies more sewage systems (“Socio-economic drivers of water pollution” section, Figs. S2, S3). People will become richer (higher GDP per capita, Fig. S3). People can afford more meat in their diets, increasing N and P intake per capita. This will lead to more N and P excretion per capita and thus more N and P in sewage systems. People can afford more cars, leading to more microplastics in sewage systems from car tire wear. In contrast, higher human development (HDI > 0.785, Fig. S2 is expected to decrease the infection rate of *Cryptosporidium*, reducing its inputs to rivers (Figs. 4, 5).

Implementing sustainable solutions for clean water requires international efforts in all 23 countries draining into the Black Sea. Already today, several international initiatives exist to protect the Black Sea (e.g., http://www.blacksea-commission.org/; Strokal 2021a, b). Such initiatives report on pollution levels and contribute to policy discussions. Our study may support future decision-making by indicating the sources of multiple pollutants in urban rivers, and the hotspots in future pollution (our scenarios). Our model results can support coordinated international efforts to control pollution. For example, in our Sustainable scenario, the SDGs are met, and wastewater treatment is improved. To reach this, international policies may need be to be implemented by the Black Sea countries. Examples of such international policies are the European “Green Deal” targets for zero pollution (European Commission 2021). One of the targets is a 30–50% reduction in microplastic pollution and associated waste. Integrating those targets by non-European countries may facilitate coordinated efforts to reduce water pollution. Strokal et al. (2022) showed the technical feasibility of reaching those targets for microplastics in the Black Sea. Future studies could build on our insights and develop environmental targets for the pollutants in rivers. Our model could help to explore strategies to reach those targets in the near and far future considering urbanization and socio-economic developments.

## Conclusions

Our study shows that fewer people may still result in more river pollution in the future. We explored future trends in point-source emissions of nutrients, microplastics, *Cryptosporidium*, and triclosan to 107 rivers draining into the Black Sea. Our model calculates that over half of the rivers will receive increasing amounts of multiple pollutants in the future. This is a result of socio-economic developments. In 74 out of 107 sub-basins, the population is expected to decrease by over 25% in the future. Over two-thirds of the population will be urban and the economy will increase by 9-fold by 2100 in the region. It is possible to reduce point-source pollution by 68–98% by 2100 with advanced technologies. Making this future reality will require coordinated international efforts among the countries of the Black Sea region. The five pollutants that we study are illustrative examples of the multi-pollutant problems that are caused by urban activities. Our study opens an opportunity for a multi-pollutant approach both in future research and policy.

## Supplementary Information

Below is the link to the electronic supplementary material.

(PDF 2776 kb)
