# Supplementary material for: The future of the Black Sea: More pollution in over half of the rivers
Source: Ambio. 2022 Sep 8;52(2):339–56. doi: 10.1007/s13280-022-01780-6 (PMC9453707; doi:10.1007/s13280-022-01780-6)
Supplement: Supplementary file 1 — (PDF 2776 kb) [file 13280_2022_1780_MOESM1_ESM.pdf]

Ambio

Supplementary Information

This supplementary information has not been peer reviewed.

Title: **The Future of the Black Sea: More Pollution in Over Half of the Rivers**

Text S1 Study area

The Black Sea is connected to the Azov Sea (Fig. 1). The total drainage area is 2.4 million km^2 and includes 23 countries, located in Western Europe, Eastern Europe and South-West Asia (Fig. 1). There are three largest rivers: Danube (Western Europe), Dnieper and Don (Eastern Europe). Their drainage areas are 1.7 million km^2, which is 70% of the total drainage area of the Black Sea. The drainage areas of the other rivers range from 2 thousand km^2 to 78 thousand km^2.

The drainage area is divided into 107 sub-basins (Strokal et al. 2019). The drainage area of the Danube River has eight sub-basins (Fig. 1). The drainage areas of the Dnieper and Don rivers have five sub-basins. The drainage areas of the other rivers are considered as individual sub-basins that discharge water directly into the Black Sea or Azov Sea.

Box S1. Model description to quantify annual inputs of pollutants to rivers of sub-basins from sewage systems and open defecation. Variables are explained in Table S1. Sources for the model inputs are given in Fig. S1 and Table S2

$RS_{i.sb}$

$$RS_{i.sb} = RS_{dir.i.sb} + RS_{sew.i.sb} \quad \text{Eq. S1}$$

$$RS_{dir.i.sb} = RS_{diru.i.sb} + RS_{dirr.i.sb} \quad \text{Eq. S2}$$

$$RS_{sew.i.sb} = RS_{sewu.i.sb} + RS_{sewr.i.sb} \quad \text{Eq. S3}$$

$$RS_{diru.i.sb} = Pop_{urb.sb} \cdot fr_{pop.dir.urb.sb} \cdot WShw_{cap.i.sb} \quad \text{For Cry, N and P} \quad \text{Eq. S4}$$

$$RS_{dirr.i.sb} = Pop_{rur.sb} \cdot fr_{pop.dir.rur.sb} \cdot WShw_{cap.i.sb} \quad \text{For Cry, N and P} \quad \text{Eq. S5}$$

$$RS_{diru.i.sb} = [Pop_{urb.sb} \cdot fr_{pop.dir.urb.sb} \cdot WShw_{cap.i.sb}] \cdot FE_{dir.i.sb} \quad \text{For DIN, DON, DIP and DOP} \quad \text{Eq. S6}$$

$$RS_{dirr.i.sb} = [Pop_{rur.sb} \cdot fr_{pop.dir.rur.sb} \cdot WShw_{cap.i.sb}] \cdot FE_{dir.i.sb} \quad \text{For DIN, DON, DIP and DOP} \quad \text{Eq. S7}$$

$$RS_{sewu.i.sb} = Pop_{urb.sb} \cdot fr_{pop.sew.urb.sb} \cdot WShw_{cap.i.sb} \cdot (1 - hw_{frem.i.sb}) \quad \text{For Cry, N, P, MP and TCS} \quad \text{Eq. S8}$$

$$RS_{sewr.i.sb} = Pop_{rur.sb} \cdot fr_{pop.sew.rur.sb} \cdot WShw_{cap.i.sb} \cdot (1 - hw_{frem.i.sb}) \quad \text{For Cry, N, P, MP and TCS} \quad \text{Eq. S9}$$

$$RS_{sewu.i.sb} = [Pop_{urb.sb} \cdot fr_{pop.sew.urb.sb} \cdot WShw_{cap.i.sb} \cdot (1 - hw_{frem.i.sb})] \cdot FE_{pnt.i.sb} \quad \text{For DIN, DON, DIP and DOP} \quad \text{Eq. S10}$$

$$RS_{sewr.i.sb} = [Pop_{rur.sb} \cdot fr_{pop.sew.rur.sb} \cdot WShw_{cap.i.sb} \cdot (1 - hw_{frem.i.sb})] \cdot FE_{pnt.i.sb} \quad \text{For DIN, DON, DIP and DOP} \quad \text{Eq. S11}$$

$WShw_{cap.i.sb}$

$$WShw_{cap.cry.sb} = I_{cry.sb} \cdot Exc_{cry} \quad \text{Eq. S12}$$

$$I_{cry.sb} = 0.05\ (5\%)\ \text{for developed sub-basins}\ (HDI_{sb} \geq 0.785) \quad \text{Eq. S13}$$

$$I_{cry.sb} = 0.10\ (10\%)\ \text{for developing sub-basins}\ (HDIsb < 0.785) \quad \text{Eq. S14}$$

$$WShw_{cap.N.sb} = [4 + 17 \cdot (GDPpppsb/68673)0.3] \cdot 0.365 \quad \text{Eq. S15}$$

$$WShw_{cap.P.hum.sb} = 1/6 \cdot WShw_{cap.N.sb} \quad \text{Eq. S16}$$

$$WShw_{cap.P.det.sb}\ \text{- given (Table S3, Fig. S5)} \quad \text{Eq. S17}$$

$$WShw_{cap.MP.tyres.sb} = 0.18\ \text{for developed sub–basins}\ (HDI_{sb} \geq 0.785) \quad \text{Eq. S19}$$

$$0.018\ \text{for developing sub–basins}\ (HDI_{sb} < 0.785) \quad \text{Eq. S20}$$

$WShw_{cap.MP.pcp.sb}$, $WShw_{cap.MP.dst.sb}$, $WShw_{cap.MP.ldry.sb}$, $WShw_{cap.TCS.sb}$ — *Given (Table S3)*

$hw_{frem.i.sb}$

$$hw_{frem.cry.sb} = \text{based on the distribution of treatment types and removal efficiencies for Cry (Table S2)}$$

$$hw_{frem.N.sb} = \text{based on the distribution of treatment types and removal efficiencies for N (Table S2)}$$

$$hw_{frem.P.sb} = \text{based on the distribution of treatment types and removal efficiencies for P (Table S2)}$$

$$hw_{frem.MP.sb} = \text{based on the } hw_{frem.P.sb}\ \text{(Table S2)}$$

$$hw_{frem.TCS.sb} = \text{based on the } hw_{frem.P.sb}\ \text{(Table S2)}$$

Table S1 Description of the model variables in Box S1. Sources for the model inputs are given in Fig. S1 and Table S2

Abbreviation	Description	Unit
i = pollutant<br>sb = sub-basins<br>Pollutants: Cryptosporidium (cry), nitrogen (N), phosphorus (P), dissolved inorganic nitrogen (DIN), dissolved organic nitrogen (DON), dissolved inorganic phosphorus (DIP), dissolved organic phosphorus (DOP), microplastics (MP) and triclosan (TCS)		
$RS_{i.sb}$	annual inputs of pollutant i to rivers of sub-basins sb from all sources (sewage systems and open defecation)	Cry: oocyst/year<br>N, P, DIN, DON, DIP, DOP: kg/year<br>MP: kg/year<br>TCS: g/year
$RS_{dir.i.sb}$	annual inputs of pollutant i to rivers of sub-basin sb from open defecation resulted from all population	
$RS_{diru.i.sb}$	annual inputs of pollutant i to rivers of sub-basin sb from open defecation resulted from urban population	
$RS_{dirr.i.sb}$	annual inputs of pollutant i to rivers of sub-basin sb from open defecation resulted from rural population	
$RS_{sew.i.sb}$	annual inputs of pollutant i to rivers of sub-basin sb from sewage systems resulted from all population	
$RS_{sewu.i.sb}$	annual inputs of pollutant i to rivers of sub-basin sb from sewage systems resulted from urban population	
$RS_{sewr.i.sb}$	annual inputs of pollutant i to rivers of sub-basin sb from sewage systems resulted from rural population	
$Pop_{urb.sb}$	urban population in sub-basin sb	People/year
$Pop_{urb.sb}$	rural population in sub-basin sb	
$Fr_{pop.dir.urb.sb}$	the fraction of urban population experiencing open defecation in sub-basin sb	0-1
$Fr_{pop.dir.rur.sb}$	the fraction of rural population experiencing open defecation in sub-basin sb	
$Fr_{pop.sew.urb.sb}$	the fraction of urban population connected to sewage systems in sub-basin sb	
$Fr_{pop.sew.urb.sb}$	the fraction of rural population connected to sewage systems in sub-basin sb	
$WShw_{cap.i.sb}$	the excretion or consumption rate of pollutant i in sub-basin sb	Cry: oocyst/cap/year<br>N, P, DIN, DON, DIP, DOP: kg/cap/year<br>MP: kg/cap/year<br>TCS: g/cap/year
$WShw_{cap.cry.sb}$	the excretion rate of cryptosporidium in human waste in sub-basin sb	
$WShw_{cap.N.sb}$	the excretion rate of nitrogen in human waste in sub-basin sb	
$WShw_{cap.P.hum.sb}$	the excretion rate of phosphorus in human waste in sub-basin sb	
$WShw_{cap.P.det.sb}$	the consumption rate of phosphorus detergents in sub-basin sb	
$WShw_{cap.MP.tyres.sb}$	the consumption rate of microplastics from car tyres in sub-basin sb	
$WShw_{cap.MP.pcp.sb}$	the consumption rate of microplastics from personal care products in sub-basin sb	
$WShw_{cap.MP.dst.sb}$	the consumption rate of microplastics from household dust in sub-basin sb	
$WShw_{cap.MP.ldry.sb}$	the consumption rate of microplastics from laundry in sub-basin sb	

$WShw_{cap.TCS.sb}$	the consumption rate of triclosan in sub-basin sb	
$I_{cry.sb}$	The infection rate in sub-basin sb	0-1
Exc_{cry}	Excretion rate per ill person	oocyst/cap/year
$GDPppp_{sb}$	Gross domestic product at purchasing power parity for sub-basin sb	2005 US$/cap/year
HDI_{sb}	Human development index for sub-basin sb	0-1
$hw_{frem.i.sb}$<br>$hw_{frem.cry.sb}$<br>$hw_{frem.N.sb}$<br>$hw_{frem.P.sb}$<br>$hw_{frem.MP.sb}$<br>$hw_{frem.TCS.sb}$	the fraction of removed pollutant i during treatment in sub-basin sb<br>the fraction of removed *Cryptosporidium* during treatment in sub-basin sb<br>the fraction of removed nitrogen during treatment in sub-basin sb<br>the fraction of removed phosphorus during treatment in sub-basin sb<br>the fraction of removed microplastics during treatment in sub-basin sb<br>the fraction of removed triclosan during treatment in sub-basin sb	0-1
$FE_{dir.i.sb}$<br>$FE_{sew.i.sb}$	the export fraction of pollutant i (only for N and P) entering rivers in form DIN, DON, DIP and DOP from open defecation<br>the export fraction of pollutant i (only for N and P) entering rivers in form DIN, DON, DIP and DOP from sewage systems	0-1

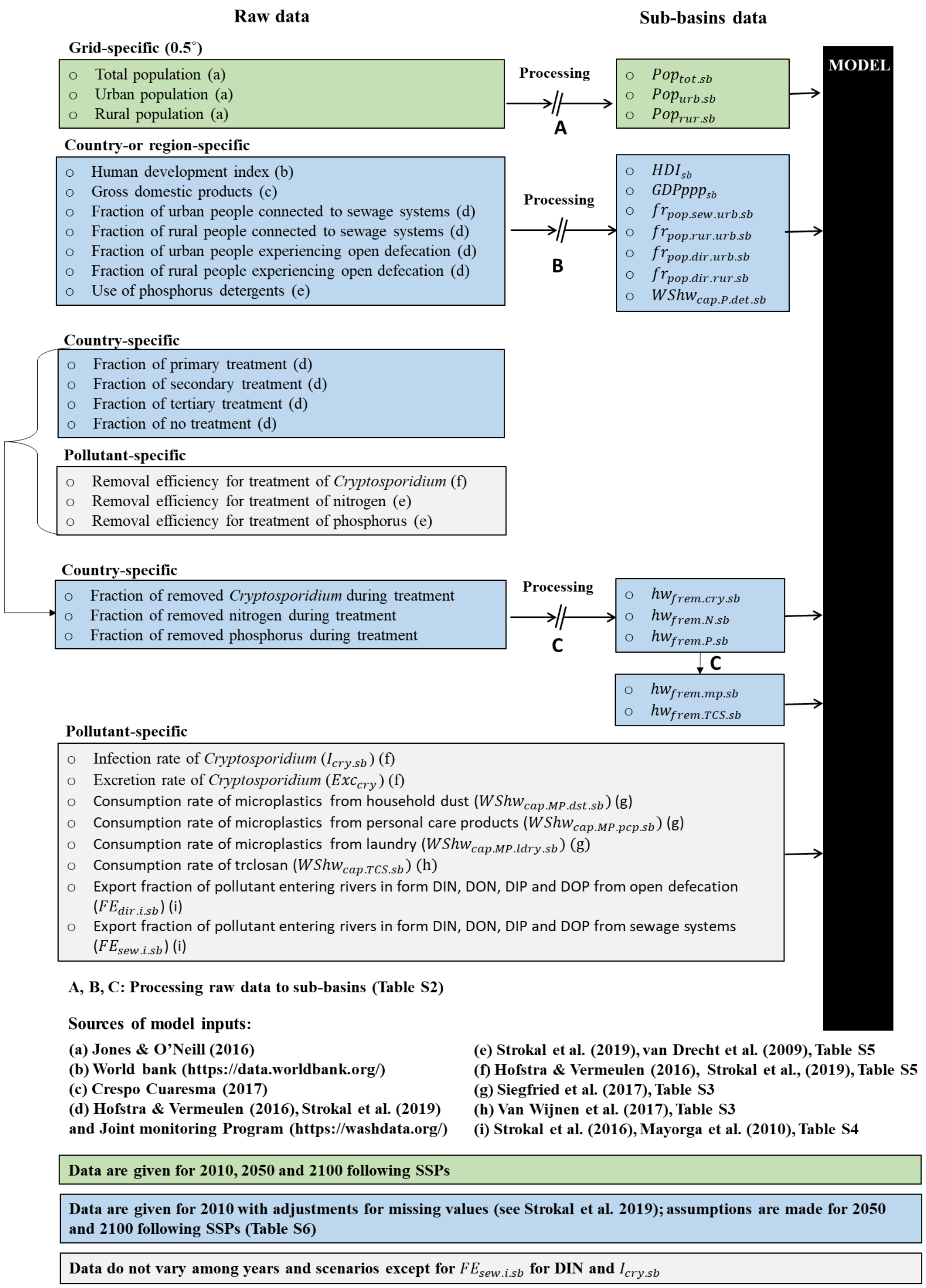

Fig. S1. Sources of model inputs. Abbreviations are referred to the equations in Box S1. Abbreviations are explained in Table S1. Table S2 provides explanation on processing the raw data to sub-basins. SSP is short for Shared Socio-economic Pathways.

Table S2 Processing the raw data to sub-basins (see Fig. S1 for the sources). Model inputs of Strokal et al. (2019), Strokal et al. (2021b), Strokal et al. (2021a) are aggregated to sub-basins.

Fig.S1	Raw data		Processing	Processed data	
	**Variable**	**Units**		**Explanation for processing**	**Units after processing**
A	Population (total, urban, rural)	People/year	Grid 0.5˚to sub-basin	Gridded values are summed over the grids covering sub-basins	People/year
B	Human development index (HDI)	0-1	Country to sub-basin	Population is used. Four steps are performed:<br>1. Country values are assigned to grids of 0.5˚. Every grid has the value of a country to which this grid belongs to;<br>2. Gridded values are multiplied by the population per grid of 0.5˚ (people/year) to get the totals;<br>3. Total values per grid are summed over the grids for each sub-basin;<br>4. Total values from step 3 are divided by the total population of each sub-basin (people/year) to get HDI for each sub-basin.<br>This approach is used for HDI in 2010, 2050 and 2100. Fig. S3 shows HDI for the sub-basins.	0-1
	Gross domestic products (GDP)	US$/cap/year	Country to sub-basin	Population is used. Four steps are performed (similar to HDI above):<br>1. Country values are assigned to grids of 0.5˚ as for HDI (see above);<br>2. Gridded values (US$/cap/year) are multiplied by the population per grid of 0.5˚ (people/year) to get the total GDP per grid (US$/year);<br>3. Total values per grid are summed over the grids for each sub-basin (US$/year for each sub-basin);<br>4. Total values from step 3 (US$/year for each sub-basin) are divided by the total population of each sub-basin (people/year) to get the GDP in US$/cap/year for each sub-basin.	US$/cap/year

				Fig S5 shows the phosphorus detergent use for the sub-basins in 2010. For 2050 and 2100, the use of detergents is assumed to be zero as a result of implemented policies that forbit the use of detergents.	
	Fraction of urban and rural people connected to sewage systems	0-1	Country to sub-basin	Four steps are performed for urban population:<br>1. Country values (0-1) are assigned to grids of 0.5° as for HDI (see above);<br>2. Gridded values (0-1) are multiplied by the population per grid of 0.5° (people/year) to get the number of urban people connected to sewage per grid (people/year);<br>3. Total values (people/year) per grid are summed over the grids for each sub-basin to get the number of urban people connected to sewage per sub-basin (people/year);<br>4. The number of urban people connected to sewage per sub-basin (people/year) is divided by the total population per sub-basin (people/year) to get the fraction of urban people connected to sewage per sub-basin.<br>The same steps are performed for rural people.	0-1
	Fraction of urban and rural people experiencing open defecation	0-1	Country to sub-basin	The same approach as for the fraction of urban and rural people connected to sewage systems, but for open defecation	kg/cap/year
	Use of phosphorus detergents	kg/cap/year	Region to sub-basin	The same approach as for GDP, but for phosphorus detergents	
C	Fraction of removed *Cryptosporidium*, nitrogen, phosphorus, microplastics and triclosan during treatment	0-1	Country to sub-basin	Six steps are performed for *Cryptosporidium*:<br>1. The fraction of removed *Cryptosporidium* is calculated for each country as (0-1):<br>(the fraction of primary treatment in each country * removal efficiencies of primary treatment) +<br>(the fraction of secondary treatment in each country * removal efficiencies of secondary treatment) +<br>(the fraction of tertiary treatment in each country * removal efficiencies of tertiary treatment) +<br>(the fraction of no treatment in each country * removal efficiencies of no treatment);<br>2. Country values from step 1 (0-1) are assigned to grids of 0.5° as for HDI (see above);	

3. Gridded values from step 2 (0-1) are multiplied by the amount of *Cryptosporidium* in human waste that goes to sewage systems per grid (oocysts/year, see step 5.3) to get the amount of *Cryptosporidium* that is treated per grid (oocysts/year);
4. Gridded values from step 3 are summed over the grids for sub-basins to get the amount of *Cryptosporidium* that is treated per sub-basin (oocysts/year);
5. The amount of *Cryptosporidium* in human waste that goes to sewage systems per sub-basins is calculated as:
   5.1 The excretion rate per sub-basin ($WShw_{cap.cry.sb}$, oocysts/cap/year) is calculated using Eq. (S12) of Box S1
   5.2 Sub-basin values from step 5.1 are assigned to grids of 0.5° similar to HDI above
   5.3 Gridded values from step 5.2 (oocysts/cap/year) are multiplied by the total people connected to sewage systems per grid (people/year) to get the amount of *Cryptosporidium* in human waste that goes to sewage systems per grid (oocysts/year). Total people connected to sewage systems per grid is calculated similar to the approach for urban and rural people as described above;
   5.4 Gridded values from step 5.3 (oocysts/year) are summed over the grids for sub-basins to get the total amount of *Cryptosporidium* in humna waste that goes to sewage systems
6. The fraction of removed *Cryptosporidium* during treatment per sub-basin is calculated by dividing the amount of *Cryptosporidium* that is treated per sub-basin (oocysts/year, step 4) by the amount of *Cryptosporidium* in humna waste that goes to sewage systems per sub-basin (oocysts/year, step 5).

The same approach is applied for nitrogen and phosphorus as for *Cryptosporidium*.

Different approach is applied microplastics and triclosan.
Here, we apply the approach of Siegfried et al. (2017) for microplastics and of van Wijnen et al. (2017) for triclosan, but for sub-basins. The known removal rates of phosphorus for sub-basins are the basis to assume the removal rates for microplastics and triclosan. Based on existing studies (Thompson et al. 2005; Heidler & Halden 2007; Dann & Hontela 2011; von der Ohe et al. 2011; Butler et al. 2012), the removal

			rates of phosphorus are related to the removal rates of microplastics and triclosan.<br>Tables S2a and S2b provide the results for removal rates.

Table S2a. Removal rates for treating microplastics in wastewater in sub-basins following the approach of (Siegfried et al. 2017). Removal rates are assigned to microplastics based on the known removal rates for phosphorus. This is inspired by the processes that drive treatments of both phosphorus and microplastics according to existing literatures (Thompson et al. 2005; Heidler & Halden 2007; Dann & Hontela 2011; von der Ohe et al. 2011; Butler et al. 2012).

Known phosphorus removal rates per sub-basin ($hw_{frem.P.sb,}$ 0-1)	Related removal rates of microplastics per sub-basin ($hw_{frem.MP.sb,}$ 0-1)
0-0.10	0
0.10-0.40	0.25
0.40-0.60	0.50
0.60-0.80	0.75
0.80-1.00	0.95

Table S2b. Removal rates for treating triclosan in wastewater in sub-basins following the approach of (van Wijnen et al. 2017). Removal rates are assigned to triclosan in a similar way as for microplastics (see Table S2a).

Known phosphorus removal rates per sub-basin ($hw_{frem.P.sb,}$ 0-1)	Related removal rates of triclosan per sub-basin ($hw_{frem.TCS.sb,}$ 0-1)
0-0.20	0
0.20-0.80	0.60
0.80-1.00	0.90

Table S3. Model inputs for excretion and consumption rates of the pollutants in the sub-basins of the Black Sea. HDI is the human development index. GDP is the gross domestic product. Source: the sub-basin scale MARINA-Global model (see the model and scenario description in the "Materials and methods" section in the main manuscript). See Fig. S1 for the sources of the information.

**Pollutants**	**Units**	**Excretion or consumption rates**	**Vary among (in grey)**		**Fig.**
			**Sub-basins**	**Years**	
Cryptosporidium	oocysts/cap/year	Eq. (S12-S14) in Box S1	grey	grey	Fig. S2 for HDI
Nitrogen<br>Phosphorus	kg/cap/year<br>kg/cap/year	Eq. (S15-S16) in Box S1	grey	grey	Fig. S3 for GDP
Phosphorus detergents	kg/cap/year	Eq. (S17) in Box S1	grey	grey	Fig. S4
Microplastics					
○ Car tyres	kg/cap/year	Eq. (S19-S20) in Box S1	grey	grey	Fig. S2 for HDI
○ Household dust<br>○ Laundry<br>○ Personal care products	kg/cap/year<br>kg/cap/year<br>kg/cap/year	0.08<br>0.12<br>0.0071			
Triclosan	g/cap/year	0.5			

Table S4. Model inputs for the export fraction of nitrogen and phosphorus entering rivers in form DIN, DON, DIP and DOP from open defecation ($FE_{dir.i.sb,}$ 0-1) and sewage systems ($FE_{sew.i.sb,}$ 0-1, Box S1). DIN and DON are dissolved inorganic and organic nitrogen, respectively. DIP and DOP are dissolved inorganic and organic phosphorus, respectively.

	**DIN**	**DON**	**DIP**	**DOP**	**Source (Fig. S1)**
$FE_{dir.i.sb}$	0.70	0.30	0.70	0.30	(Strokal et al. 2016)
$FE_{sew.i.sb}$	Calculated*	0.14	1.00	0.01	(Mayorga et al. 2010)**

*It varies among the years and scenarios. It is calculated using the approach of (Mayorga et al. 2010; Strokal et al. 2016) as follows:

$FE_{sew.DIN.sb} = a + b * (hw_{frem.N.sb} / hw_{frem.N.max})$

Where,

$FE_{sew.DIN.sb}$ is the export fraction of nitrogen (N) entering rivers in form DIN from sewage systems in sub-basin sb (0-1). $hw_{frem.N.sb}$ is the fraction of removed nitrogen (N) during treatment in sub-basin sb (0-1). $hw_{frem.N.max}$ is the maximum removal rate for nitrogen (N) among (0-1). It is set at 0.88 (88% of N removed in human waste, corresponds to the advanced treatment). a and b are coefficients. a equals to 0.485 and b equals to 0.255.

** $hw_{frem.N.max}$ is adjusted to the maximum removal efficiency that is used in this study (88%).

Table S5. Removal efficiencies of pollutants during wastewater treatment.

Pollutant	Treatment type				Source (Fig. S1)
	Primary	Secondary	Tertiary	No treatment	
Cryptosporidium	0.10	0.55	0.98	0.00	(Hofstra & Vermeulen 2016; Strokal et al. 2019; Strokal et al. 2021b)
Nitrogen	0.10	0.42	0.88	0.00	(Van Drecht et al. 2009; Strokal et al. 2019; Strokal et al. 2021b)
Phosphorus	0.10	0.51	0.95	0.00	

Table S6. Assumptions for the three scenarios for the years 2050 and 2100. In this study, country values are processed to sub-basin specific model inputs (see Box S1, Fig S1 and Table S2). HDI is human development index. GDP is gross domestic products. BAU is Business as Usual. Source for the country-specific assumptions: (Strokal et al. 2021b). SSP is shared Socio-economic Pathway (see Fig. S1 for the sources).

**Model inputs (Box S1)**	**BAU[a]**		**Economy[a]**		**Sustainability[a]**	
	**2050**	**2100**	**2050**	**2100**	**2050**	**2100**
Total people	SSP2	SSP2	SSP5	SSP5	SSP1	SSP1
Urbana people	SSP2	SSP2	SSP5	SSP5	SSP1	SSP1
Rural people	SSP2	SSP2	SSP5	SSP5	SSP1	SSP1
GDP	SSP2	SSP2	SSP5	SSP5	SSP1	SSP1
HDI	10% increase 2010-2050	10% increase 2050-2100	20% increase 2010-2050	20% increase 2050-2100	As Economy	As Economy
Connection rate of people to sewage (% reduced gap)						
○ Urban	20% reduced gap 2010-2050*	50% reduced gap 2050-2100*	30% reduced gap 2010-2050*	60% reduced gap 2050-2100*	As in Economy	As in Economy
○ Rural	5% reduced gap 2010-2050*	20% reduced gap 2050-2100*	10% reduced gap 2010-2050*	30% reduced gap 2050-2100*	As in Economy	As in Economy
People experiencing open defecation	Stopped	Stopped	Stopped	Stopped	Stopped	Stopped
Distribution of treatment types (% shift to the next treatment type between 2010 and 2050)**						
○ HDI≥0.785	shift 50% 2010-2050	shift 70% 2050-2100	As BAU	As BAU	All tertiary	All tertiary
○ HDI<0.785	shift 30% 2010-2050	shift 50% 2050-2100	As BAU	As BAU	1/2 secondary and 1/2 tertiary	All tertiary
Use of phosphorus detergents	Forbidden	Forbidden	Forbidden	Forbidden	Forbidden	Forbidden

* Example for 2010 and 2100: 40% of the population with sewage connections in 2010. A gap would be is 60% (100% - 40%). Reducing a gap of 50%: (60% gap * 50% reduction)/100% = 30%. This means that 30% of the people will be connected to sewage systems as a result of the reduced gap. The total percentage of people connected to sewage systems for 2100 = % people connected to sewage systems in 2010 (40%) + % people

connected to sewage systems as a result of the reduced gap (30%). This gap closure approach was implemented in the Millennium Ecosystem Assessment scenarios (Seitzinger et al. 2010). ** The order for the shift in treatment types: no treatment, primary, secondary and tertiary treatment. Details are in (Strokal et al. 2021b).

a: The study of Strokal et al. (2021b) reflects BAU as a moderate scenario for urbanization. The Economy scenario is reflected as a scenario with high urbanization and moderate levels of wastewater removals in (Strokal et al. 2021b). The sustainability scenario is reflected as a scenario with high urbanization and high levels of wastewater removals in (Strokal et al. 2021b).

Table S7. References and remarks of the other studies that are used to compare with our model results.

Letters from Table 1	References and remarks
**a**	Global NEWS (Nutrient Export from Watersheds) (Van Drecht et al. 2009; Mayorga et al. 2010; Strokal & Kroeze 2013). The model provides values for 2000 and takes the basin modelling approach. In the model, 63 basins are included for the Black Sea region. The values in this table are the sum of the point-source inputs of nutrients to rivers from all 63 basins.
**b**	SWAT (Soil and Water Assessment Tool) model (Malagó et al. 2017). The values in this table are for nitrogen and phosphorus discharges in the Danube River basin using the SWAT model. The values are the ranges for the period of 1995-2009.
**c**	IMAGE-GNM (Global Nutrient Model) for point and diffuse sources (Beusen et al. 2015). The model provides values for the period of 1900-2000 globally and regionally. We use the values for 2000 in this comparison. Beusen et al. (2015) provide values for nitrogen and phosphorus inputs to all rivers of the Black Sea and Mediterranean Sea regions. These values are around 4 TgN/year and around 0.8 TgP/year for the year 2000. Around 12% of the nutrient inputs to rivers of the Black Sea and Mediterranean Sea are from sewage systems. Using this, we estimated 0.48 TgN/year and 0.09 TgP/year entered rivers of the Black Sea and Mediterranean Sea regions in 2000.
**d**	IMAGE-GNM (Global Nutrient Model) for point-source inputs of nitrogen and phosphorus to rivers from sewage systems (van Puijenbroek et al. 2019). The model provides values for the period of 1970-2050 globally and regionally. We use the values for 2010 in this comparison. van Puijenbroek et al. (2019) provide values only for the world regions. The Black Sea region is part of the two regions: (1) Western and Central Europe and (2) Russia and Central Asia. The values in the table are the range for these two regions.
**e**	Microplastics model for point-source river export of microplastics to the European sea including the Black Sea (Siegfried et al. 2017). The model provides values for 2000 and future. For this comparison, we took the river export of microplastics to the Black Sea for 2000. We estimated inputs to all rivers of the Black Sea region using the data on retentions in rivers and river export from Siegfried et al. (2017).<br>- Siegfried et al. (2017) estimated 4 kton of microplastics entered the Black Sea from the rivers considering retentions of microplastics due to water consumption (fraction of 0.07 in Table S1 of Siegfried et al. (2017)) and sedimentation. For the retentions due to sedimentation, we estimated an average of 0.75 (for small basins) and 0.9 (for large basins) indicated in Table 2 of Siegfried et al. (2017). This average is 0.83. Then we estimated inputs of microplastics to rivers from point sources as follows: river export of microplastics from point sources (4 kton in 2000) / the export fraction of microplastics (0-1). The export fraction is estimated as (1-0.07)*(1-0.85). This is according to the approach of Siegfried et al. (2017).<br>- We estimated inputs to Danube rivers from point sources using river export and retentions from Siegfried et al. (2017). We did this as follows:

	river export of microplastics from Danube (1503 ton/year) / the export fraction of microplastics (0-1). The export fraction is estimated as (1-0.03)*(1-0.85). 0.03 is the fraction related to removal of microplastics from rivers via water consumption. This fraction is from Mayorga et al. (2010), which is also used in Siegfried et al. (2017). 0.85 reflects the retention of microplastics in rivers due to sedimentation according to Siegfried et al. (2017). 0.85 is an average of 0.75 (small basins) and 0.90 (large basins). We took an average because our study has sub-basins in different sizes. These retentions are from Siegfried et al. (2017)
**f**	A study that combines measured values to estimate the amount of plastics in the river mouth of Danube (Lechner et al. 2014). According to this study, 1533 ton of plastics entered the Black Sea from Danube. We estimated inputs of plastics to rivers of the Danube basin using the approach of Siegfried et al. (2017) as: river export (1533 ton/year) / the export fraction. The export fraction is (1-0.03)*(1-0.85) (see the footnote for (a) above).
**g**	TCS model for river export of triclosan from sewage systems (van Wijnen et al. 2017). It takes a basin scale modelling approach. Our study is based on this approach, but adjusted for 2010 and sub-basins following the Shared-socio-economic pathways.

Table S8. Set up of the sensitivity analysis to test the sensitivity of the model outputs to changes in model inputs. We selected six main model inputs that influence inputs of all five pollutants to rivers: *Cryptosporidium*, nitrogen, phosphorus, triclosan and microplastics. We changed the model inputs for 107 sub-basins of the Black Sea region for 2010.

Model inputs	Changes in model inputs
1. Urban population	+10%
2. Rural population	+10%
3. Fraction of urban people connected to sewage systems	+10%
4. Fraction of rural people connected to sewage systems	+10%
5. Treatment efficiencies*	+10%
6. Excretion rates of pollutants per capita	+10%

*Removal is changed for *Cryptosporidium*, nitrogen and phosphorus. Removal of triclosan and microplastics is influenced by changes in the removal of phosphorus.

Table S9. Percentage of the sub-basin area and of the total population in the drainage area of the Black Sea for which increases in inputs of pollutants are calculated for the period of 2010-2050 and 2050-2100 for the Business as Usual (BAU), Economy and Sustainability scenarios. Source: Section 2 in the main text, Box S1, Fig. S1 and Tables S1-S8.

**Pollutants**	**2010-2050**		**2050-2100**	
	**Area (%)**	**Population (%)**	**Area (%)**	**Population (%)**
BAU				
Cryptosporidium	2	2	2	0.2
Total dissolved nitrogen	95	97	51	48
Total dissolved phosphorus	70	68	15	13
Microplastics	87	85	6	2
Triclosan	62	66	54	49
Economy				
Cryptosporidium	20	30	1	0.1
Total dissolved nitrogen	99	100	27	40
Total dissolved phosphorus	93	94	8	19
Microplastics	87	85	8	21
Triclosan	66	70	10	22
Sustainability				
Cryptosporidium	0	0	2	0.4
Total dissolved nitrogen	0.2	0.03	53	52
Total dissolved phosphorus	0	0	53	52
Microplastics	5	12	2	0.4
Triclosan	5	12	2	0.4

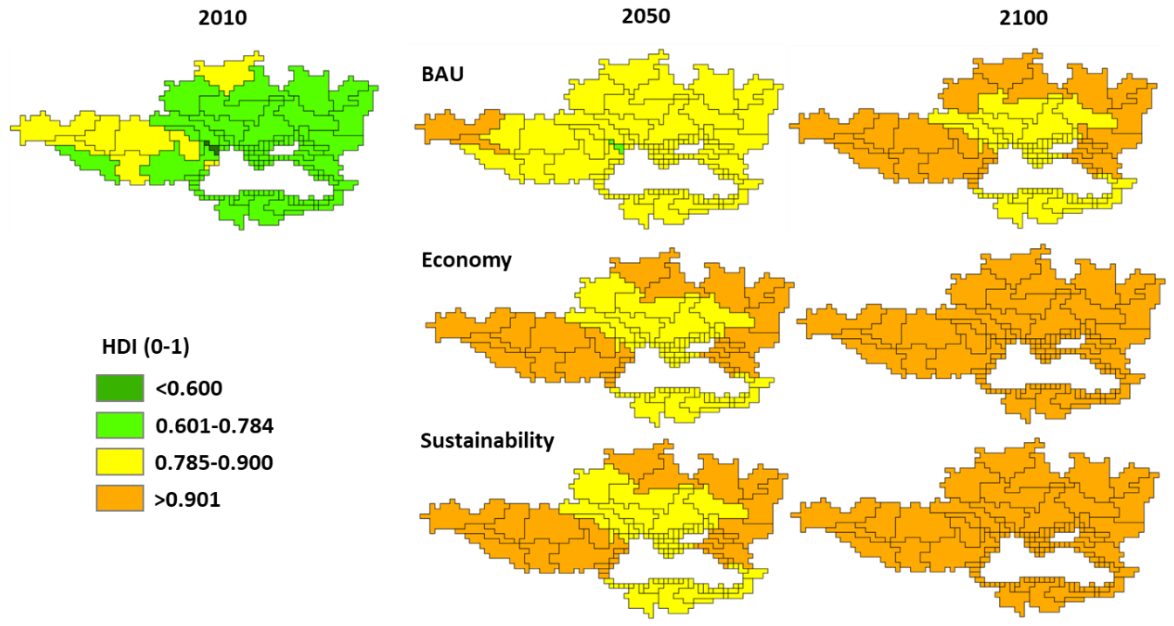

Fig. S2. Human development index (HDI) for the sub-basins of the Black Sea region in 2010, 2050 and 2100 for the Business as Usual (BAU), Economy and Sustainability scenarios (0-1). Source: the sub-basin scale MARINA-Global model (see the model and scenario description in the "Materials and methods" section in the main manuscript).

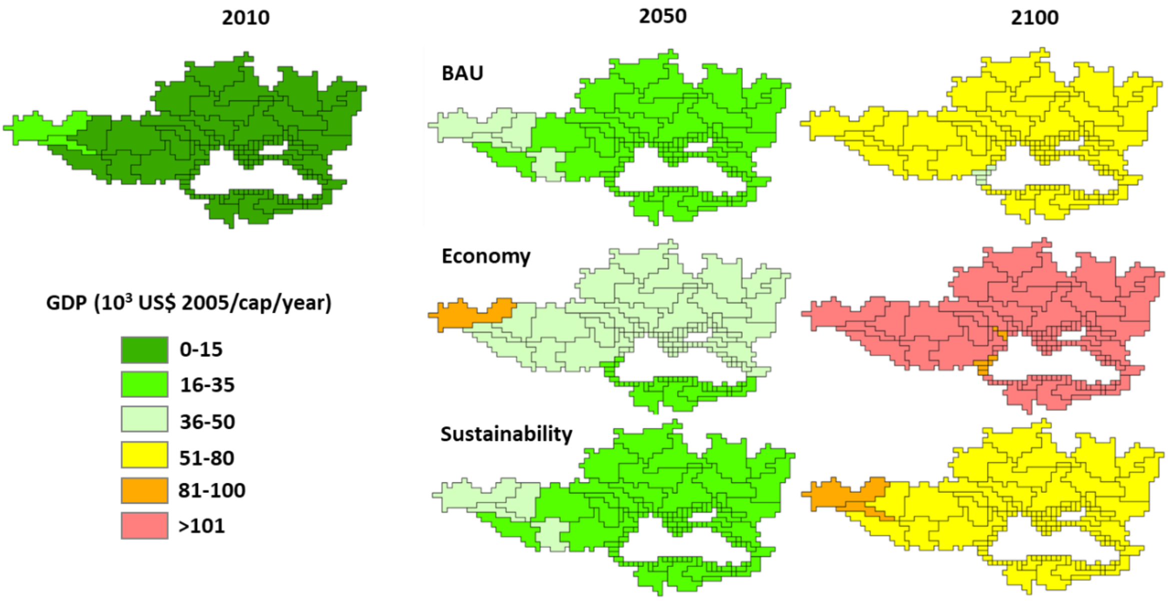

Fig. S3. Gross domestic products (GDP) for the sub-basins of the Black Sea in 2010, 2050 and 2100 for the Business as Usual (BAU), Economy and Sustainability scenarios (10^3 US$/cap/year). Source: the sub-basin MARINA-Global model (see the model and scenario description in the "Materials and methods" section in the main manuscript).

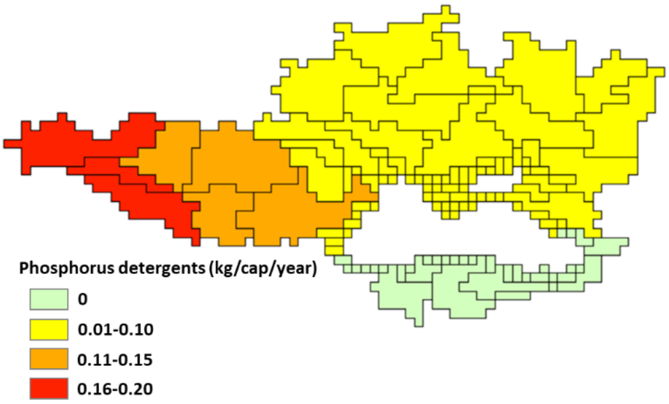

Fig. S4. Phosphorus detergents use in the sub-basins of the Black Sea in 2010 (kg/cap/year). Values for 2050 and 2100 are zero based on the scenarios. Table S2 provides the information how these sub-basins values are derived. Source: the sub-basin MARINA-Global model (see the model and scenario description in the "Materials and methods" section in the main manuscript).

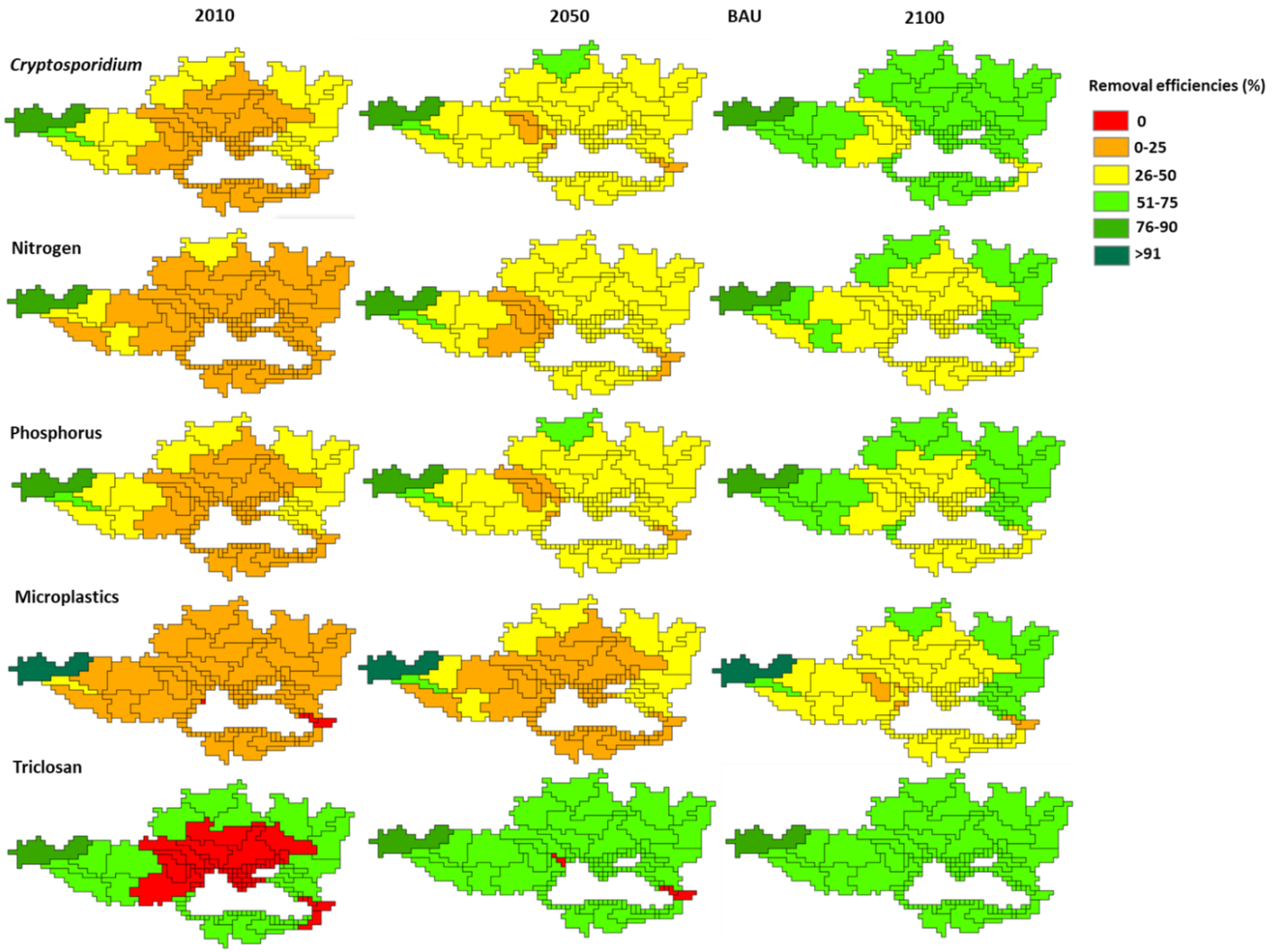

Fig. S5. Removal efficiencies of pollutants during treatment for the sub-basins of the Black Sea in 2010, 2050 and 2100 for the Business as Usual (BAU) scenario (%). Source: the sub-basin scale MARINA-Global model (see the model and scenario description in the "Materials and methods" section in the main manuscript).

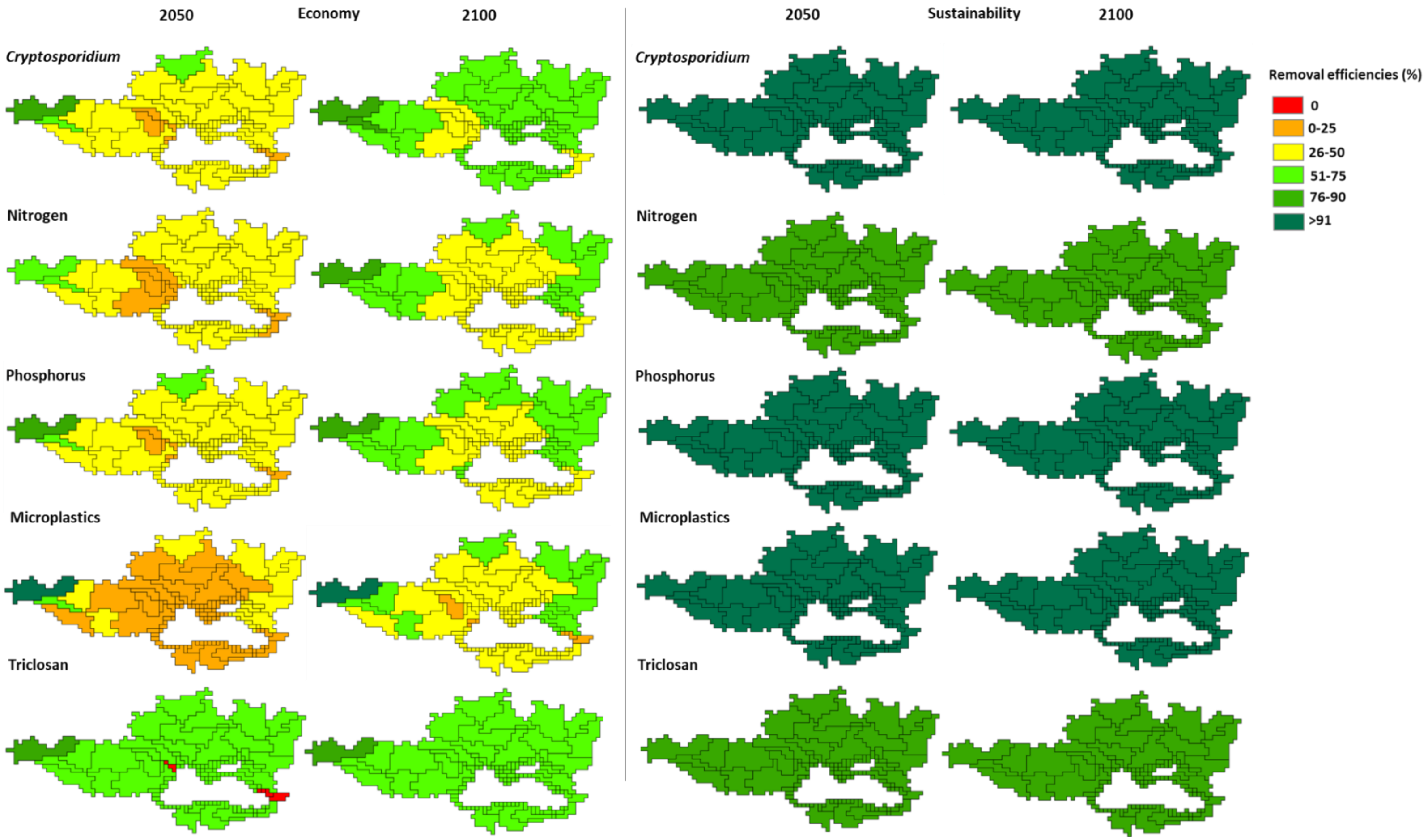

Fig. S6. Removal efficiencies of pollutants during treatment for the sub-basins of the Black Sea in 2010, 2050 and 2100 for the Economy and Sustainability scenarios (%). Source: the sub-basin scale MARINA-Global model (see the model and scenario description in the “Materials and methods” section in the main manuscript).

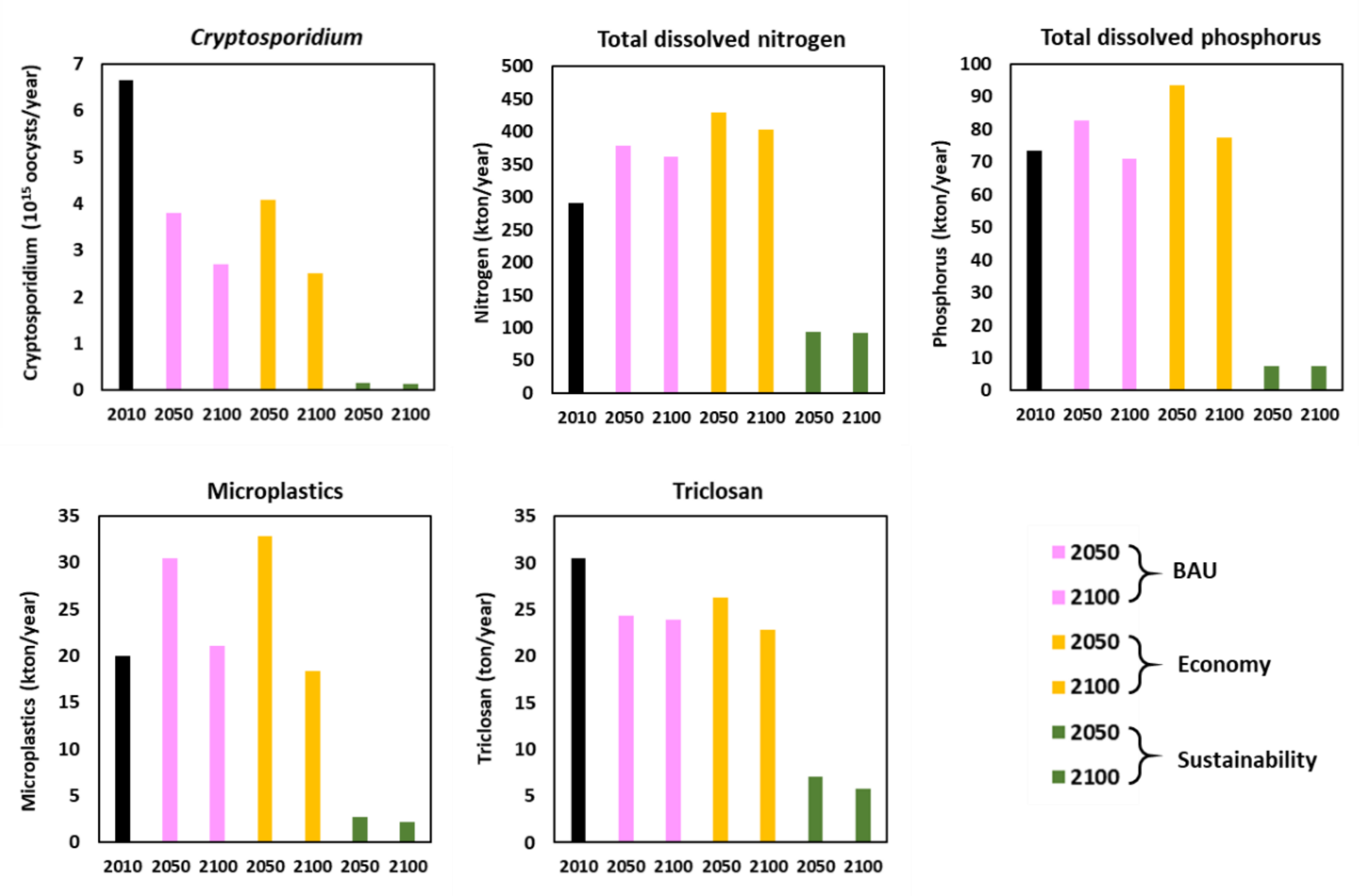

Fig. S7. Annual inputs of the five pollutants to the rivers of the Black Sea from point sources in 2010, 2050 and 2100 for the Business as Usual (BAU), Economy and Sustainability scenarios. Descriptions of the scenarios are in the "Materials and methods". Source: the sub-basin scale MARINA-Global model (see Section 2).

References used in the supporting information

1.

Beusen, A., Bouwman, A., Van Beek, L., Mogollón, J. & Middelburg, J. (2015). Global riverine N and P transport to ocean increased during the twentieth century despite increased retention along the aquatic continuum. *Biogeosciences Discussions*, 12, 20123-20148.

2.

Butler, E., Whelan, M.J., Sakrabani, R. & van Egmond, R. (2012). Fate of triclosan in field soils receiving sewage sludge. *Environmental Pollution*, 167, 101-109.

3.

Dann, A.B. & Hontela, A. (2011). Triclosan: environmental exposure, toxicity and mechanisms of action. *Journal of applied toxicology*, 31, 285-311.

4.

Heidler, J. & Halden, R.U. (2007). Mass balance assessment of triclosan removal during conventional sewage treatment. *Chemosphere*, 66, 362-369.

5.

Hofstra, N. & Vermeulen, L.C. (2016). Impacts of population growth, urbanisation and sanitation changes on global human Cryptosporidium emissions to surface water. *International Journal of Hygiene and Environmental Health*, 219, 599-605.

6.

Lechner, A., Keckeis, H., Lumesberger-Loisl, F., Zens, B., Krusch, R., Tritthart, M., Glas, M. & Schludermann, E. (2014). The Danube so colourful: A potpourri of plastic litter outnumbers fish larvae in Europe's second largest river. *Environmental Pollution*, 188, 177-181.

7.

Malagó, A., Bouraoui, F., Vigiak, O., Grizzetti, B. & Pastori, M. (2017). Modelling water and nutrient fluxes in the Danube River Basin with SWAT. *Science of The Total Environment*, 603-604, 196-218.

8.

Mayorga, E., Seitzinger, S.P., Harrison, J.A., Dumont, E., Beusen, A.H.W., Bouwman, A.F., Fekete, B.M., Kroeze, C. & Van Drecht, G. (2010). Global Nutrient Export from WaterSheds 2 (NEWS 2): Model development and implementation. *Environmental Modelling & Software*, 25, 837-853.

9.

Seitzinger, S.P., Mayorga, E., Bouwman, A.F., Kroeze, C., Beusen, A.H.W., Billen, G., Van Drecht, G., Dumont, E., Fekete, B.M., Garnier, J. & Harrison, J.A. (2010). Global river nutrient export: A scenario analysis of past and future trends. *Global Biogeochemical Cycles*, 24, GB0A08.

10.

Siegfried, M., Koelmans, A.A., Besseling, E. & Kroeze, C. (2017). Export of microplastics from land to sea. A modelling approach. *Water Research*, 127, 249-257.

11.

Strokal, M., Bai, Z., Franssen, W., Nynke, H., Koelmans, A.A., Ludwig, F., Ma, L., van Puijenbroek, P., Spanier, J.E., Vermeulen, L.C., van Vliet, M.T.H., van Wijnen, J. & Kroeze, C. (2021a). Metadate supporting a\the article "Urbanization: an increasing source of multiple pollutants to rivers in the 21st century". *Wageningen University & Research* *https://doi.org/10.17026/dans-zyx-jce3*.

12.

Strokal, M., Bai, Z., Franssen, W., Nynke, H., Koelmans, A.A., Ludwig, F., Ma, L., van Puijenbroek, P., Spanier, J.E., Vermeulen, L.C., van Vliet, M.T.H., van Wijnen, J. & Kroeze, C. (2021b). Urbanization: an increasing source of multiple pollutants to rivers in the 21st century. *Urban Sustainability*, 1, 24.

13.

Strokal, M. & Kroeze, C. (2013). Nitrogen and phosphorus inputs to the Black Sea in 1970–2050. *Regional Environmental Change*, 13, 179-192.

14.

Strokal, M., Kroeze, C., Wang, M., Bai, Z. & Ma, L. (2016). The MARINA model (Model to Assess River Inputs of Nutrients to seAs): Model description and results for China. *Science of The Total Environment*, 562, 869-888.

15.

Strokal, M., Spanier, J.E., Kroeze, C., Koelmans, A.A., Flörke, M., Franssen, W., Hofstra, N., Langan, S., Tang, T., van Vliet, M.T.H., Wada, Y., Wang, M., van Wijnen, J. & Williams, R. (2019). Global multi-pollutant modelling of water quality: scientific challenges and future directions. *Current Opinion in Environmental Sustainability*, 36, 116-125.

16.

Thompson, A., Griffin, P., Stuetz, R. & Cartmell, E. (2005). The fate and removal of triclosan during wastewater treatment. *Water environment research*, 77, 63-67.

17.

Van Drecht, G., Bouwman, A.F., Harrison, J. & Knoop, J.M. (2009). Global nitrogen and phosphate in urban wastewater for the period 1970 to 2050. *Global Biogeochemical Cycles*, 23, GB0A03.

18.

van Puijenbroek, P.J.T.M., Beusen, A.H.W. & Bouwman, A.F. (2019). Global nitrogen and phosphorus in urban waste water based on the Shared Socio-economic pathways. *Journal of Environmental Management*, 231, 446-456.

19.

van Wijnen, J., Ragas, A. & Kroeze, C. (2017). River export of triclosan from land to sea: A global modelling approach. *Science of The Total Environment*, 621, 1280-1288.

20.

von der Ohe, P.C., Dulio, V., Slobodnik, J., De Deckere, E., Kühne, R., Ebert, R.-U., Ginebreda, A., De Cooman, W., Schüürmann, G. & Brack, W. (2011). A new risk assessment approach for the prioritization of 500 classical and emerging organic microcontaminants as potential river basin specific pollutants under the European Water Framework Directive. *Science of the Total Environment*, 409, 2064-2077.
